# Combined and Distinct Roles of Agr Proteins in Clostridioides difficile 630 Sporulation, Motility, and Toxin Production

**DOI:** 10.1128/mBio.03190-20

**Published:** 2020-12-22

**Authors:** Ummey Khalecha Bintha Ahmed, Tyler M. Shadid, Jason L. Larabee, Jimmy D. Ballard

**Affiliations:** a Department of Microbiology and Immunology, The University of Oklahoma Health Sciences Center, Oklahoma City, Oklahoma, USA; Harvard Medical School

**Keywords:** *Clostridioides difficile*, Agr, TcdA, Cas9, autoinducing peptide, *Clostridium difficile*, TcdB, sporulation

## Abstract

The Clostridioides difficile accessory gene regulator 1 (*agr1*) locus consists of two genes, *agrB1* and *agrD1*, that presumably constitute an autoinducing peptide (AIP) system. Typically, AIP systems function through the AgrB-mediated processing of AgrD to generate a processed form of the AIP that provides a concentration-dependent extracellular signal. Here, we show that the C. difficile 630 Agr1 system has multiple functions, not all of which depend on AgrB1. CRISPR-Cas9n deletion of *agrB1*, a*grD1*, or the entire locus resulted in changes in transcription of sporulation-related factors and an overall loss in spore formation. Sporulation was recovered in the mutants by providing supernatant from stationary-phase cultures of the parental strain. In contrast, C. difficile motility was reduced only when both AgrB1 and AgrD1 were disrupted. Finally, in the absence of AgrB1, the AgrD1 peptide accumulated within the cytoplasm and this correlated with increased expression of *tcdR* (15-fold), as well as *tcdA* (20-fold) and *tcdB* (5-fold), which encode the two major C. difficile toxins. The combined deletion of *agrB1*/*agrD1* or deletion of only *agrD1* did not significantly alter expression of *tcdR* or *tcdB* but did show a minor effect on *tcdA* expression. Overall, these data indicate that the Agr1-based system in C. difficile 630 carries out multiple functions, some of which are associated with prototypical AIP signaling and others of which involve previously undescribed mechanisms of action.

## INTRODUCTION

Clostridioides difficile encodes autoinducing peptide (AIP)-based quorum sensing activities similar to the well-studied Agr system in Staphylococcus aureus (1). The prototypical Agr system includes adjacent genes that encode a sensor histidine kinase (AgrC), response transcriptional regulator (AgrA), protease (AgrB), and the signaling peptide (AgrD). During bacterial growth, AgrD is processed by AgrB into a thiolactone-containing AIP that accumulates outside the cell. As the AIP extracellular concentration increases, the peptide interacts with and triggers autophosphorylation of AgrC, which subsequently phosphorylates AgrA as part of a two-component kinase pathway. AgrA then mediates the downstream transcriptional effects associated with this quorum signaling system (2–5). Interestingly, several pathogenic *Clostridia* possess a bicistronic operon encoding only AgrB and AgrD (6), raising the possibility that the two-gene system has adapted distinct functions independent of the sensor kinase and response regulator.

In C. difficile, three forms of the Agr system resembling the prototypical system in S. aureus have been identified (7–9). Only one system, Agr1, has been found in all sequenced strains of C. difficile (7). Agr2, which has been found in C. difficile strain R20291 (ribotype 027), resembles S. aureus Agr and includes all four *agr* genes of a complete Agr system, but the four genes are arranged in an opposite order to that of S. aureus (see Fig. S1 in the supplemental material). Agr3, which has been found in C. difficile ribotype 078, is carried by a C. difficile-specific bacteriophage (8) and contains a three-gene system lacking the AgrA response regulator (Fig. S1). Agr1, the focus of our study, is a two-gene locus encoding only AgrB1 and AgrD1 and is the only AIP system present in the clinically relevant C. difficile 630 strain (7–9). No cognate histidine kinases or response regulators have been identified as AgrC1 or AgrA1. One plausible explanation for the lack of *agrC1* and *agrA1* associated with the *agr1* loci is that the genes are present elsewhere on the genome of C. difficile. Alternatively, AgrB1 and AgrD1 may function in ways that do not require a two-component histidine kinase signaling system. Indeed, the absence of AgrA3 in the Agr3 system also suggests that Agr systems may have been adapted for other regulatory functions in C. difficile.

10.1128/mBio.03190-20.1 FIG S1 Variations in *agr* locus among C. difficile strains. The length of the intervening/overlapping sequences between genes and the size of the proteins encoded by the *agr* locus genes are color-coded in green and blue, respectively. Sequence similarities among AgrB1/AgrD1 of *agr1* and other Agr systems are marked in red. Download FIG S1, TIF file, 1.4 MB. Copyright © 2020 Ahmed et al. 2020 Ahmed et al. This content is distributed under the terms of the Creative Commons Attribution 4.0 International license.

Despite lacking AgrC1 and AgrA1, the Agr1 system appears to contribute to C. difficile virulence. Using allelic exchange, Darkoh and colleagues generated AgrB1D1-negative forms of C. difficile strain 630 and R20291 (7, 10). The AgrB1D1 mutants in both strains lost the ability to transcribe *tcdA* and *tcdB*, the genes encoding two major C. difficile toxins. The virulence of the AgrB1D1 mutant was also reduced as measured in a murine model of C. difficile infection. Thus, the two-gene Agr1 system is important for C. difficile pathogenesis, but how it influences virulence factor gene expression in the absence of a two-component system is unclear.

Since the *agr1* operon is important for C. difficile virulence but does not encode a histidine kinase and response regulator, we decided to examine individual *agrB1*, *agrD1*, and *agrB1D1* mutants for changes in expression of important regulators associated with toxin production, motility, and sporulation. We reasoned that if Agr1 functions like a typical Agr system, then genetic deletion of *agrB1* or *agrD1* should result in a phenotype identical to that of an *agrB1D1* mutant, because in each instance no signaling peptide would be released into the extracellular environment. In addition, identifying the key regulators that connect Agr with virulence factor expression should increase our understanding of this system in C. difficile. We first developed a Cas9 nickase system for precision DNA editing in C. difficile and used this to generate gene deletions in *agrB1*, *agrD1*, and *agrB1D1*. Results from transcriptional and phenotypic analyses of these mutants suggest that Agr1 influences sporulation efficiency and requires the combined activities of AgrB1 and AgrD1. In contrast, and unexpectedly, the data show that C. difficile toxin expression is impacted only in the absence of AgrB1 and corresponds to intracellular accumulation of the AgrD1 peptide. These findings indicate that Agr1 influences C. difficile activities through both AgrB1-dependent and AgrB1-independent mechanisms.

## RESULTS

### Deletion of the *agr1* genes in C. difficile 630.

To explore the possible individual roles of the two Agr1 proteins, precise deletions of the *agrB1* and *agrD1* genes were generated in C. difficile 630 using a CRISPR-Cas9 nickase gene editing system (detailed in Materials and Methods). In addition to the individual deletions of *agrB1* and *agrD1*, this system was also used to delete the entire *agr1* locus in order to compare our mutants to *agr1* mutants created in previous studies (7, 10).

**FIG 1:**
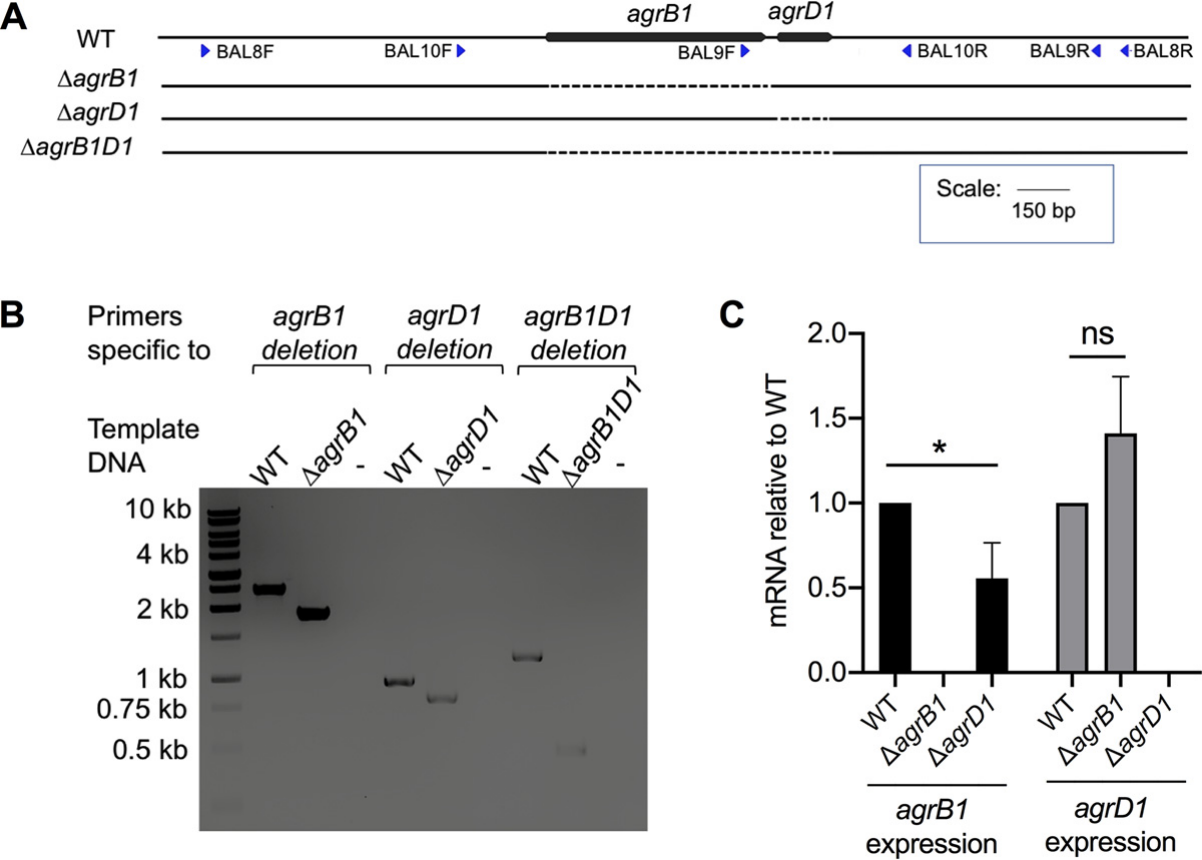
Deletion of *agr1* locus genes in C. difficile. (A) *agr1* locus in the wild type (WT) and *agr1* mutants. Blue arrows indicate positions of the screening primers, and dashed lines depict the deleted region in the respective mutants. (B) PCR screening of potential *agr1* mutant clones. (C) Relative abundance of *agrB1* and *agrD1* in the *agrB1* and *agrD1* mutants. The means and standard error of the means from three biological replicates are shown, with significance being determined by the two-tailed Student *t* test. *, *P* ≤ 0.05; ns, nonsignificant.

In Fig. 1A, the arrangement of the *agr1* locus is depicted along with the design of the *agrB1* deletion (Δ*agrB1*), the *agrD1* deletion (Δ*agrD1*), and the *agr1* locus deletion (Δ*agrB1D1*). To confirm each deletion in C. difficile 630, genomic DNA was PCR amplified using primers that span *agrB1*, *agrD1*, or the entire *agrB1D1* open reading frame. As shown in Fig. 1B, Δ*agrB1* was confirmed by a decrease in the PCR product size from 2,448 bp to 1,887 bp (primers: BAL8F and BAL8R), and Δ*agrD1* was confirmed by a reduction in PCR product size from 946 bp to 799 bp (primers: BAL9F and BAL9R). Finally, Δ*agrB1D1* was detected by a decrease in the PCR product size from 1,197 bp to 454 bp (primers: BAL10F and BAL10R) (Fig. 1B and Table S1).

10.1128/mBio.03190-20.7 TABLE S1 List of primers used in this study. Download Table S1, PDF file, 0.1 MB. Copyright © 2020 Ahmed et al. 2020 Ahmed et al. This content is distributed under the terms of the Creative Commons Attribution 4.0 International license.

Next, we examined each mutant for polar effects by measuring transcript levels of *agrB1* and *agrD1* in both of the single gene deletions. As shown in Fig. 1C, as expected, no *agrB1* transcripts were observed in the Δ*agrB1* strain but *agrD1* transcripts were present at a level similar to those in C. difficile 630 (wild type [WT]). *agrD1* transcripts were not detected in C. difficile Δ*agrD1*, and *agrB1* transcripts were reduced approximately 2-fold compared to those in the parent C. difficile 630 strain (Fig. 1C). Additionally, the C. difficile Δ*agrB1*, Δ*agrD1*, and Δ*agrB1D1* mutants were examined by whole-genome sequencing to determine if off-target effects were introduced by the CRISPR-Cas9n gene editing system. In addition to the expected deletions, one gene loss/fusion event in the *agrD1* mutant and only one or two single nucleotide polymorphisms (SNPs) were found in each of the mutants. Two SNPs were found in C. difficile Δ*agrB1*, one of which is intergenic (CD630_28280/CD630_28290) and the another of which results in one amino acid change (S49Y) in the *pstI* (phosphoenolpyruvate-protein phosphotransferase) gene. The observed gene loss and fusion event in the Δ*agrD1* mutant occurred in the *ermB1*/*ermB* locus, which is bacteriophage encoded. The putative hydrolase CD630_20080, which is encoded in between a gene duplication, *ermB1-CD630_20080-ermB*, is absent from the genome for the Δ*agrD1* mutant, and the prior duplication of *ermB1*/*ermB* is now a single *ermB* gene. The SNP observed in the Δ*agrD1* mutant is also intergenic (*agrD1*/CD630_27490). In C. difficile Δ*agrB1D1*, insertion of a single guanine (G) was found in the *fliF* (flagellar M-ring protein) gene (position 1474), which resulted in a premature stop codon resulting in a predicted FliF protein with a 25-amino-acid truncation.

### C. difficile *agr1* mutants differentially express key regulators of sporulation, motility, and toxin production.

**FIG 2:**
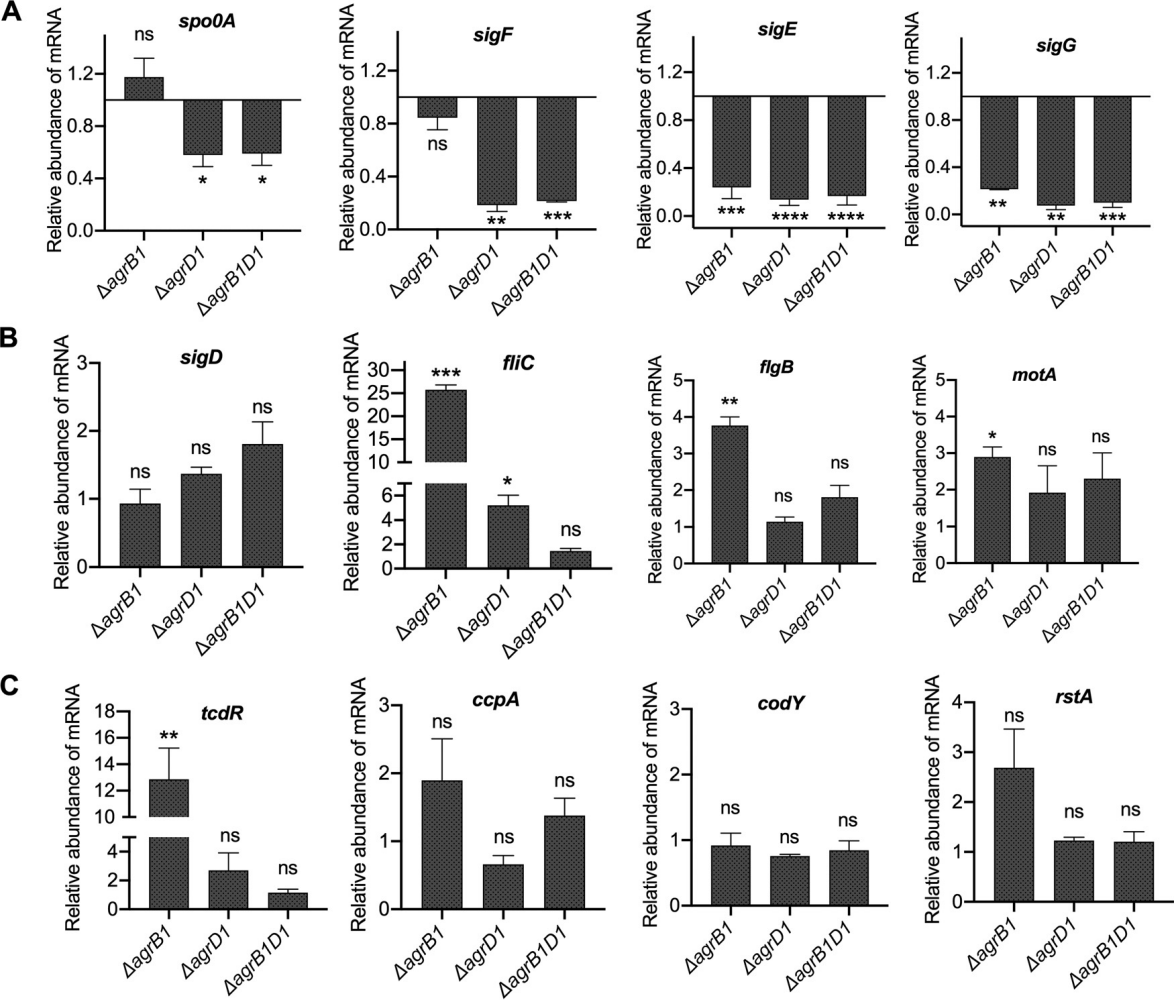
Altered expression of sporulation-, motility-, and toxin-associated genes in the *agr1* mutants. Shown are results of RT-qPCR analysis of genes involved in the regulation of sporulation (A), motility (B), and toxin production (C) in the WT, Δ*agrB1*, Δ*agrD1*, and Δ*agrB1D1* strains from early stationary phase grown in BHIS medium. Data are presented as means ± SEMs from at least three independent biological replicates, with significance being determined by the two-tailed Student *t* test. *, *P* ≤ 0.05.

The Agr system in many Gram-positive bacteria, including some *Clostridia*, regulates a wide range of cellular processes, including sporulation and toxin production (11–14). Therefore, to begin with, we examined Agr1 mutants for the expression of a set of genes that are known to be involved in the regulation of sporulation, motility, and toxin production in C. difficile. Sporulation is regulated through Spo0A, the master regulator of sporulation, and by alternative sigma factors corresponding to *sigE*, *sigF*, and *sigG*. The transcript level of *spo0A* was unchanged in C. difficile Δ*agrB1* but was significantly decreased in both the C. difficile Δ*agrD1* and Δ*agrB1D1* mutants. Spo0A-mediated downstream regulation of its target genes (*sigE*, *sigF*, and *sigG*) depends on the phosphorylation of Spo0A and does not always correlate with its transcript level (15–17). Therefore, we next examined the C. difficile *agr1* mutants for the expression of phosphorylated-Spo0A target genes *sigE*, *sigF*, and *sigG*. As shown in Fig. 2A, *sigF* transcripts were significantly decreased in the C. difficile Δ*agrD1* and Δ*agrB1D1* mutants, with a slight decrease in C. difficile Δ*agrB1*. All three mutants exhibited significantly lower transcript levels of *sigE* and *sigG*.

To elucidate the connection, if any, between the Agr1 system and motility in C. difficile *agr1* mutants, we next examined expression levels of flagellar alternative sigma factor gene *sigD* and other *sigD*-regulated genes in the *agr1* mutants. As shown in Fig. 2B, no change in *sigD* transcripts was observed, but we discovered altered transcript levels of *sigD*-dependent genes *fliC* (flagellin), *flgB* (flagellar rod protein), and *motA* (one of the motor proteins). In C. difficile Δ*agrB1*, transcripts of all three *sigD*-dependent genes were elevated, with *fliC* exhibiting the most substantial increase (∼26-fold [Fig. 2B]). Examination of C. difficile Δ*agrD1* revealed a 5-fold increase in *fliC* transcript levels, while *flgB* and *motA* transcripts remained unchanged. Transcript levels of *fliC*, *flgB*, and *motA* were unchanged in C. difficile Δ*agrB1D1* (Fig. 2B).

Previous studies have shown a connection between the Agr1 system and toxin production in C. difficile (7, 10). However, the regulatory network that connects the Agr1 system to toxin production is unknown. Therefore, we examined transcription of a number of known regulators of toxin production, including TcdR (toxin-specific sigma factor), CcpA (catabolite control protein), CodY, and RstA (regulator of sporulation and toxins) (18–22). As shown by quantitative reverse transcription-PCR (RT-qPCR), the transcript levels of *tcdR* were elevated ∼13-fold in C. difficile Δ*agrB1* but remained unchanged in the C. difficile Δ*agrD1* and Δ*agrB1D1* mutants (Fig. 2C). No significant differences were observed in the transcript levels of *ccpA*, *codY*, or *rstA* in any of the mutants (Fig. 2C).

### C. difficile *agr1* mutants are defective in sporulation.

**FIG 3:**
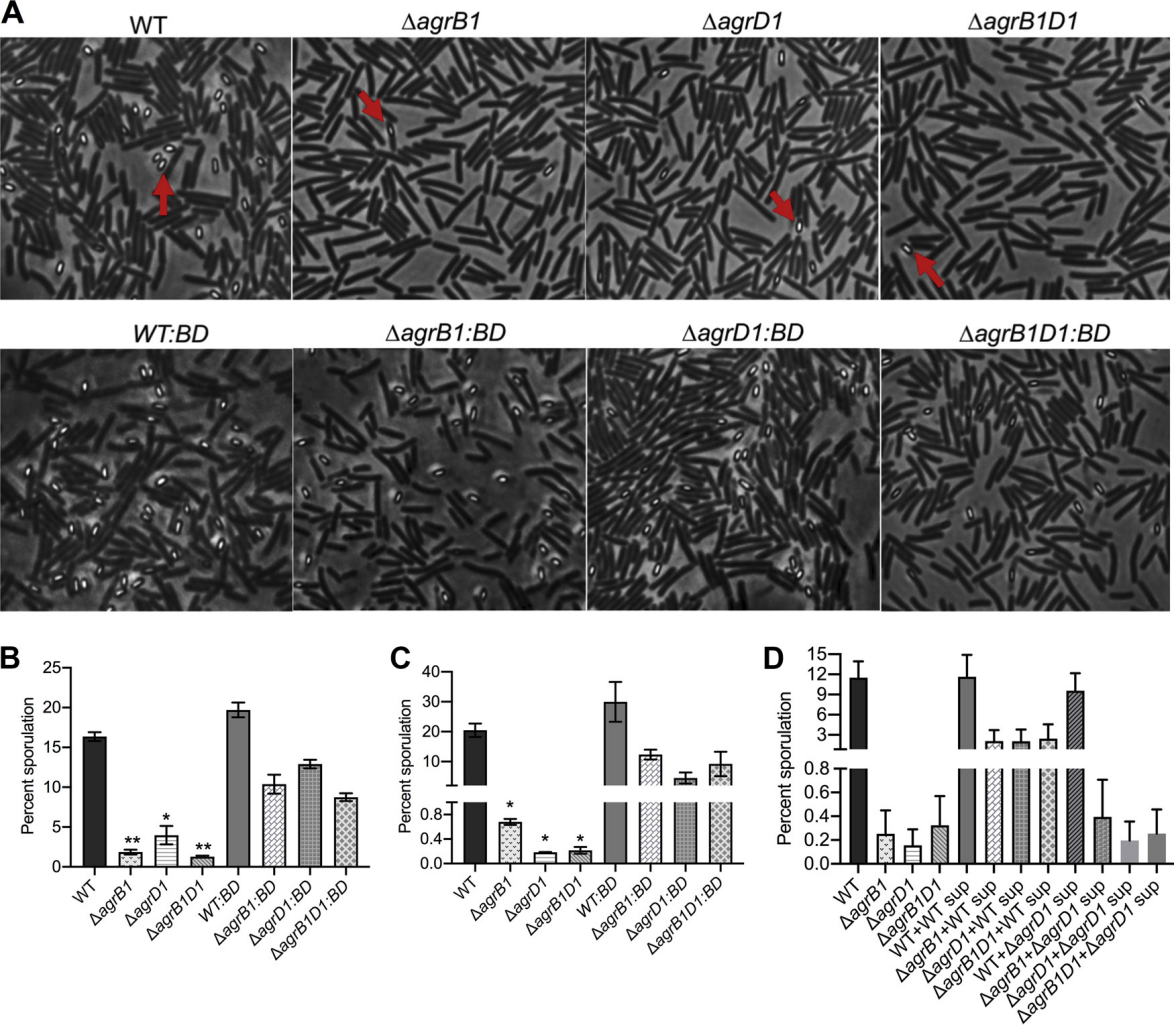
Sporulation is significantly decreased in the *agr1* mutants. (A) Phase-contrast microscopy of WT, Δ*agrB1*, Δ*agrD1*, Δ*agrB1D1*, *WT*::*BD*, Δ*agrB1*::*BD*, Δ*agrD1*::*BD*, and Δ*agrB1D1*::*BD* strains grown on 70:30 agar at 20 h. Red arrows point to spores. (B) Percent sporulation of the WT, *agr1* mutants, and *agr1* complement strains grown on 70:30 plates was calculated from phase-contrast microscopy at 20 h. The means and standard error of the means from three biological replicates are shown, with significance being determined by the two-tailed Student *t* test. (C) Percent sporulation of the WT, *agr1* mutants, and *agr1* complement strains grown on 70:30 plates was calculated by heat resistance assay at 22 h. The means and standard error of the means from two biological replicates are shown, with significance being determined by the two-tailed Student *t* test. *, *P* ≤ 0.05. (D) Percent sporulation of the WT and *agr1* mutants on 70:30 plates supplemented with either WT or Δ*agrD1* culture supernatant. Results are presented as the means and standard errors of the means from two biological replicates.

The expression of several sporulation-associated genes was reduced in all three *agr1* mutants (Fig. 2A). Therefore, in our next set of experiments, we determined if sporulation was altered by growing the *agr1* mutants for 20 h on sporulation medium and then quantifying percent sporulation by phase-contrast microscopy. Results from this experiment revealed a sporulation efficiency of 16.4% for the parental C. difficile 630 strain, while the C. difficile Δ*agrB1*, Δ*agrD1*, and Δ*agrB1D1* strains showed reduced sporulations of 1.9%, 4.0%, and 1.3%, respectively (Fig. 3A and B).

To confirm that the decreased sporulation in the *agr1* mutants was attributed to the sole loss of the *agr1* genes, complement strains (C. difficile Δ*agrB1*::*BD*, strain TMS005; C. difficile Δ*agrD1*::*BD*, strain TMS006; and C. difficile Δ*agrB1D1*::*BD*, strain TMS007) were constructed by chromosomally integrating the complete *agr1* locus along with the 365-bp upstream region into each of the mutants. As a control, we also introduced the same region (365 bp + *agr1*) on the chromosome of the parental C. difficile strain to create the C. difficile *WT*::*BD* strain (strain TMS004). The expression of *agrB1* and *agrD1* was confirmed in the complement strains (Fig. S5B). Sporulation of the *agr1* mutants was partially recovered in the complemented strains. C. difficile Δ*agrB1*::*BD*, C. difficile Δ*agrD1*::*BD*, and C. difficile Δ*agrB1D1*::*BD* showed 10.4%, 12.9%, and 8.7% sporulation, respectively (Fig. 3A and B). C. difficile *WT*::*BD* demonstrated increased sporulation (19.7%) over the parental C. difficile strain (16.4%). The asporogenous phenotype still predominated when the incubations were extended another 24 h (44 h total) (Fig. S4). We further assessed sporulation using a heat resistance assay. Based on this approach, sporulation was found to be reduced to less than 1% in each of the mutants and partially recovered in the complemented strains (Fig. 3C). Based on the results from the heat resistance assay, it is likely that some of the light-refractory bodies counted visually were not viable spores. While the overall heat resistance was 20.7% in C. difficile WT, that increased to 31.8% in C. difficile *WT*::*BD*. CFU counts and percent sporulation of the WT, *agr1* mutants, and complement strains from the heat resistance assay can be found in Table S2. Overall, these results indicate that the Agr1 system is critical for C. difficile sporulation.

10.1128/mBio.03190-20.8 TABLE S2 Percent sporulation of C. difficile 630 WT, *agr1* mutant, and *agr1* complement strains at 22 h. Download Table S2, PDF file, 0.03 MB. Copyright © 2020 Ahmed et al. 2020 Ahmed et al. This content is distributed under the terms of the Creative Commons Attribution 4.0 International license.

### Supernatant from C. difficile 630 restores sporulation in the *agr1* mutants.

To determine if the extracellular peptide produced by the Agr1 system drove the observed sporulation phenotype in the *agr1* mutants, we tested the ability of early stationary-phase sterile supernatants from either C. difficile WT or C. difficile Δ*agrD1* to induce sporulation of *agr1* mutants on 70:30 sporulation agar plates. We found that adding supernatant from C. difficile WT strain resulted in 8.4-, 13.56-, and 7.53-fold-increased percent sporulation, respectively, in C. difficile Δ*agrB1*, Δ*agrD1*, and Δ*agrB1D1* strains compared to that of corresponding mutants alone (Fig. 3D). In contrast, no noticeable restoration of the sporulation phenotypes in the Agr1 mutants was found when supernatant from C. difficile Δ*agrD1* was used to treat the *agr1* mutants on 70:30 plates (Fig. 3D). These results indicate a potential role for an extracellular signal in C. difficile sporulation and further highlight the importance of the Agr1 system in C. difficile sporulation.

### The C. difficile Δ*agrB1D1* strain is nonmotile and defective in flagellum production.

**FIG 4:**
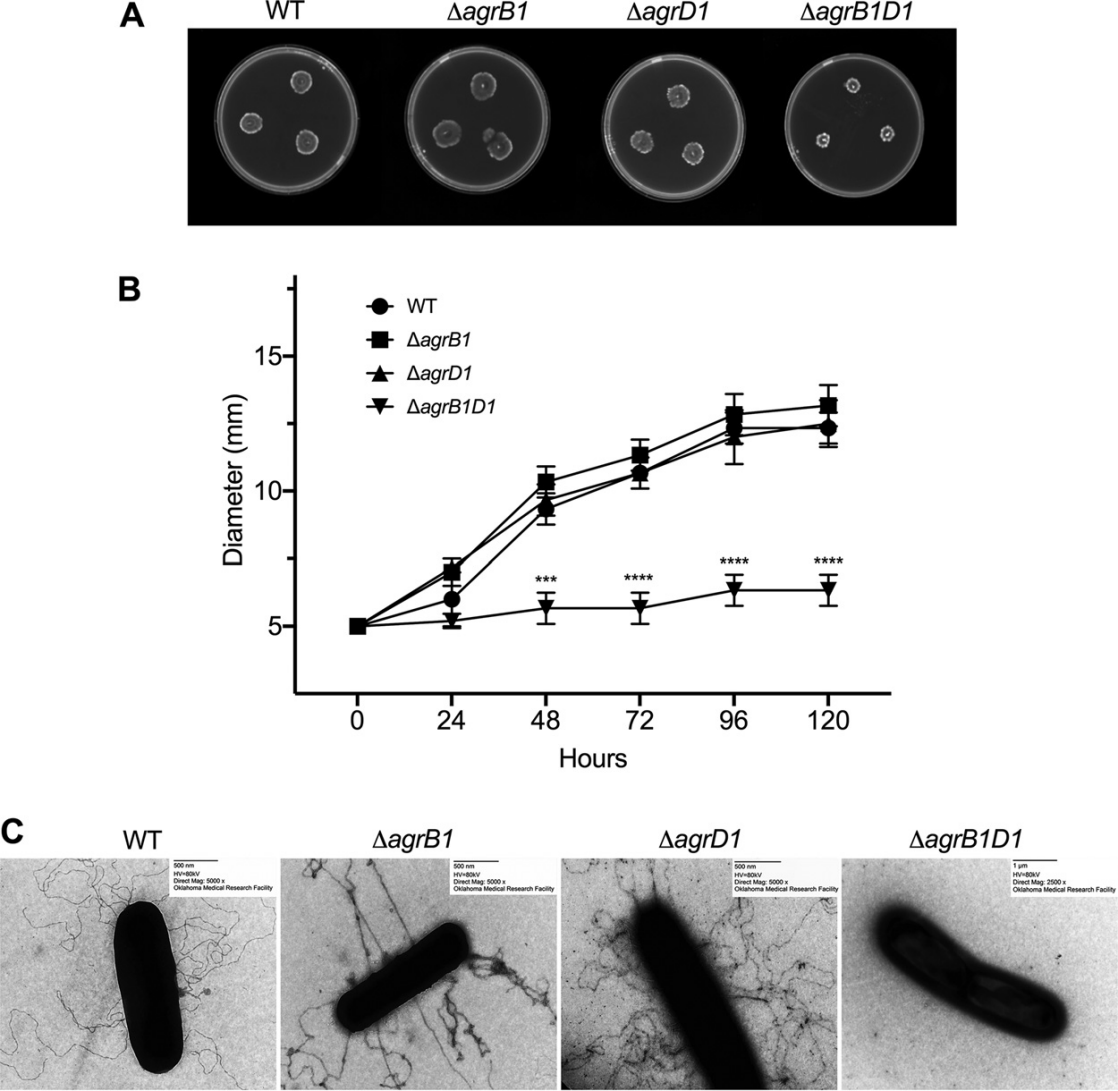
C. difficile Δ*agrB1D1* exhibits a nonmotile, aflagellate phenotype. (A) Swimming motility of the WT and *agr1* mutants in one-half concentration BHI with 0.3% agar at 120 h. (B) The swim diameters on the plate were measured every 24 h up to 120 h. (C) Negative-staining TEM images of the flagella of the WT and *agr1* mutants stained with 4% uranyl acetate. The means and standard error of the means from three biological replicates are shown, with significance being determined by the two-tailed Student *t* test. *, *P* ≤ 0.05.

Genes associated with motility and flagellum production were found to be altered in the C. difficile 630 *agr1* mutants (Fig. 2B); therefore, we next examined whether motility was affected in any of the *agr1* mutants. Swimming motility of the C. difficile *agr1* mutants was assayed on soft agar plates over 120 h, and the results revealed no significant change in motility in either the C. difficile Δ*agrB1* or Δ*agrD1* mutant compared to the parental C. difficile 630 strain (Fig. 4A and B). In C. difficile Δ*agrB1D1*, swimming motility was not detected during the 120-h time frame of the assay (Fig. 4A and B). We also examined swarming motility of the *agr1* mutants, and phenotypes similar to that of swimming motility assay were observed in case of all three mutants (data not shown).

We next visualized flagellum production by transmission electron microscopy (TEM) in each of the mutants. From this TEM assay, we found that both C. difficile Δ*agrB1* and C. difficile Δ*agrD1* produced flagella similarly to the C. difficile WT strain. In contrast, flagella were not observed in C. difficile Δ*agrB1D1* (Fig. 4C). Next, we determined if complementing C. difficile Δ*agrB1D1* (C. difficile 630 *agr*Δ*B1D1*::*BD*) could restore the nonmotile phenotype of the Δ*agrB1D1* strain. After examination of multiple clones of C. difficile Δ*agrB1D1*::*BD*, we could not rescue the nonmotile phenotype of C. difficile Δ*agrB1D1* by complementation (data not shown).

### Deletion of *agrB1* increases TcdA and TcdB production in C. difficile.

**FIG 5:**
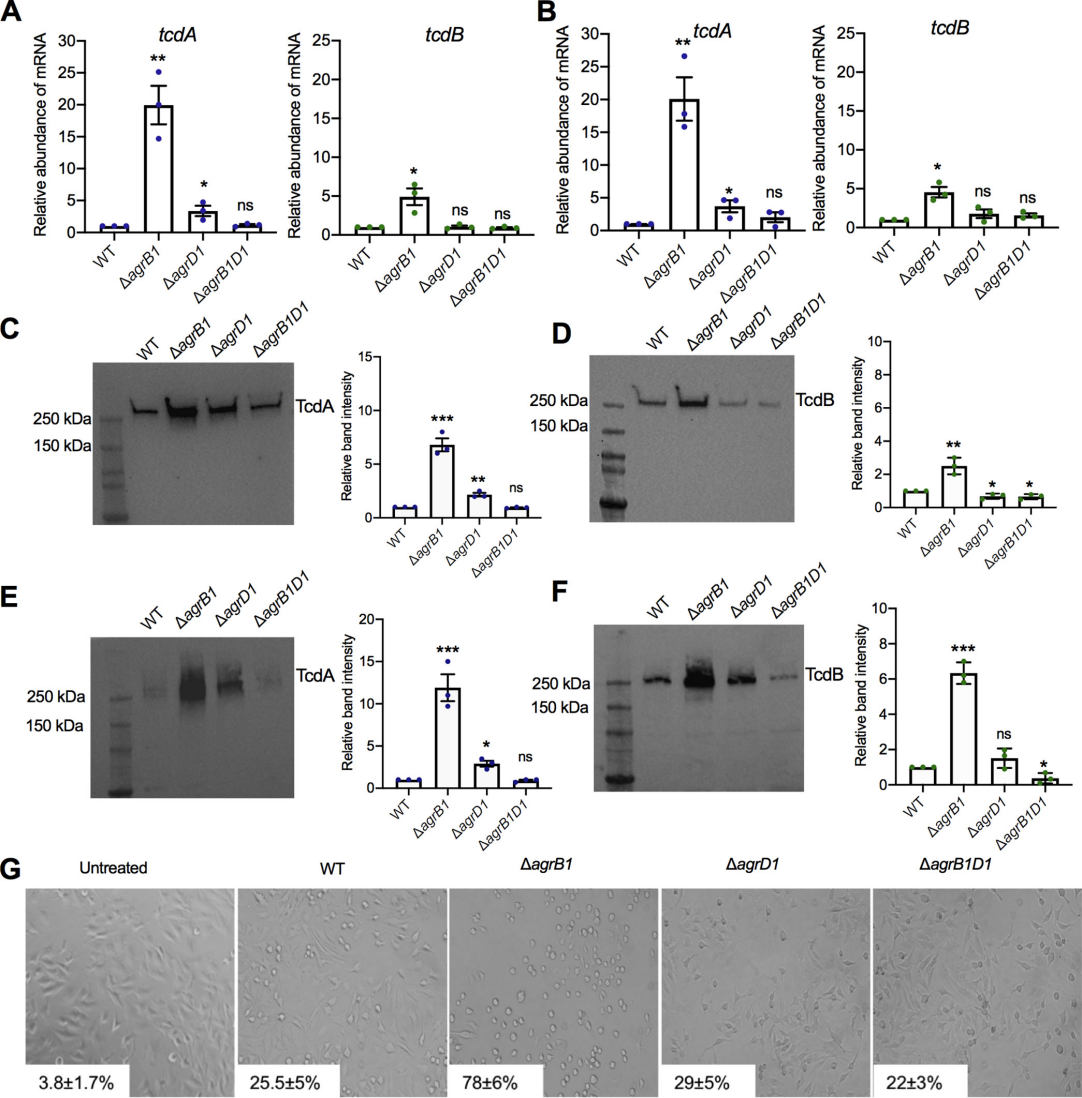
Transcript levels of *tcdA*, *tcdB*, and *tcdR* and protein levels of TcdA and TcdB are increased in Δ*agrB1*. Shown are the results of RT-qPCR analysis of *tcdA* and *tcdB* mRNA transcripts in the *agr1* mutant cultures at early stationary phase grown in BHIS (A) or BHI (B) medium and Western blot analysis of TcdA and TcdB (D) along with densitometry in Δ*agrB1*, Δ*agrD1*, and Δ*agrB1D1* cell culture at early stationary phase grown in BHIS (C and D) or BHI (E and F). Data are presented as means and SEMs of three independent biological replicates (*, *P* ≤ 0.05 using the two-tailed Student *t* test), and blots are representative of those independent experiments. (G) Representative cell rounding images showing HeLa cells treated with early-stationary-phase supernatant of WT, Δ*agrB1*, Δ*agrD1*, and Δ*agrB1D1* strains. Inset numbers show percent cell rounding as means and standard errors of the means of two biological replicates.

The Agr system has been found to regulate the expression of toxin genes in bacterial pathogens such as S. aureus and Clostridium perfringens (11–13, 23–25). Most recently, work on C. difficile 630 and C. difficile R20291 demonstrated that disruption of the *agr1* locus and concomitant loss in expression of both *agrB1* and *agrD1* resulted in the abolishment of the production of TcdA and TcdB, the two major toxins produced by most clinically relevant strains of C. difficile (10). Furthermore, as described above, we detected altered transcript levels of *tcdR* in the *agr1* mutants (Fig. 2C). Therefore, in the next set of experiments, the C. difficile 630 strains containing the individual deletions of *agrB1* or *agrD1* were examined to determine if these mutations caused altered levels of toxin production in BHIS medium (brain heart infusion medium supplemented with 5 g/liter of yeast extract). The three mutants and the wild-type C. difficile 630 strain were grown to stationary phase, and transcript levels of *tcdA* and *tcdB* were measured by RT-qPCR. As shown in Fig. 5A and B, transcript levels were unchanged in C. difficile Δ*agrB1D1*. C. difficile Δ*agrB1* exhibited higher transcript levels for both toxin genes than did C. difficile WT, with a 20-fold increase in *tcdA* and a 5-fold increase in *tcdB* (Fig. 5A). Evaluation of C. difficile Δ*agrD1* revealed a 3-fold increase in *tcdA* transcript levels, but *tcdB* transcript levels remained unchanged (Fig. 5A). Toxin levels were also analyzed by immunoblotting using C. difficile lysates taken from stationary-phase cultures. As shown in the immunoblots, TcdA was increased approximately 7-fold in C. difficile Δ*agrB1* and about 2-fold in C. difficile Δ*agrD1*, while no change was observed in C. difficile Δ*agrB1D1* (Fig. 5C). The immunoblots revealed that TcdB levels increased approximately 2.5-fold in C. difficile Δ*agrB1* and were slightly decreased in the other two mutants (Fig. 5D).

To determine if growth conditions might influence the toxin expression phenotypes of the Agr1 mutants, we also cultured the strains in BHI medium, which results in lower overall growth than with BHIS. As shown in Fig. 5B, E, and F, growing the Agr1 mutants in BHI medium resulted in toxin expression patterns similar to those detected when the mutants were grown in BHIS medium. Corresponding TGX stain-free gels that were used to develop these immunoblots are shown in Fig. S3A to D.

10.1128/mBio.03190-20.2 FIG S2 Final Cas9n vector (pTMS002/pTMS003/pTMS004) for the deletion of *agr1* genes generated in the pMTL84151 background. The modification in the backbone includes incorporation of Clostridium sporogenes *pfdx* promoter, codon-optimized *Cas9* nickase genes, P4 synthetic promoter, gRNA, and homology region. Download FIG S2, TIF file, 0.7 MB. Copyright © 2020 Ahmed et al. 2020 Ahmed et al. This content is distributed under the terms of the Creative Commons Attribution 4.0 International license.

**FIG 6:**
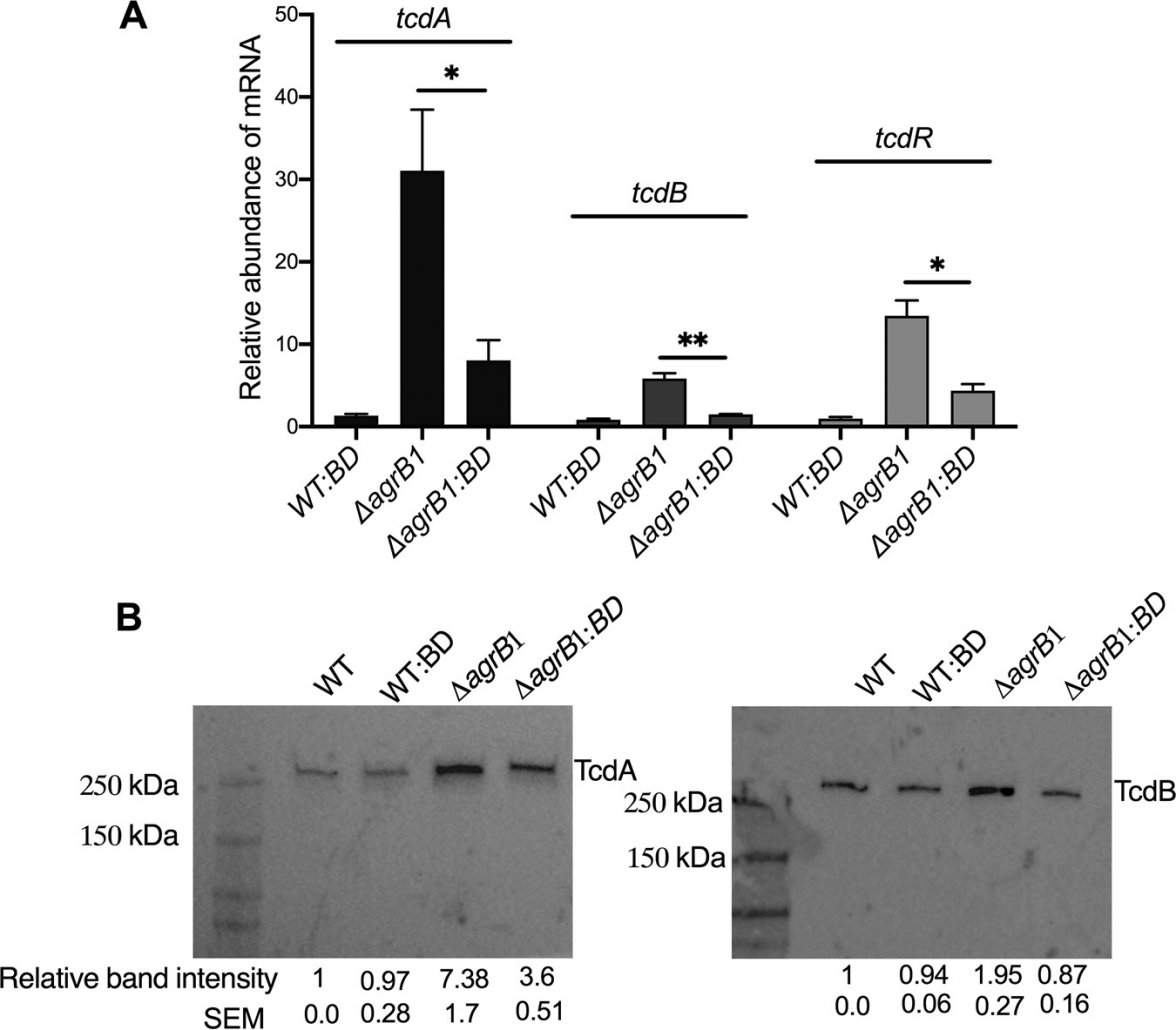
Toxin production and *tcdA*, *tcdB*, and *tcdR* gene expression are partially restored in the Δ*agrB1* complement strain. (A) RT-qPCR analysis of *tcdA*, *tcdB*, and *tcdR* in *WT*::*BD* and Δ*agrB1*::*BD* strains along with WT and Δ*agrB1* early-stationary-phase cultures. (B) Western blot analysis of TcdA and TcdB in WT, WT::*BD*, Δ*agrB1*, and Δ*agrB1*::*BD* early-stationary-phase cell cultures. Data are presented as means ± SEMs from at least three independent biological replicates, with significance being determined by the two-tailed Student *t* test. *, *P* ≤ 0.05.

10.1128/mBio.03190-20.3 FIG S3 TGX stain-free gels used to develop western blots in Fig. 5 and Fig. 6 in the main text. Total proteins were loaded on a 4 to 15% TGX stain-free gel. The image was captured using a ChemiDoc Bio-Rad imager before transferring proteins onto a PVDF membrane. Panels A, B, C, D, E, and F, respectively, correspond to the blots in Fig. 5C to F, 6B (TcdA), and 6B (TcdB). Download FIG S3, TIF file, 0.3 MB. Copyright © 2020 Ahmed et al. 2020 Ahmed et al. This content is distributed under the terms of the Creative Commons Attribution 4.0 International license.

10.1128/mBio.03190-20.4 FIG S4 Sporulation efficiency of *agr1* mutants remained significantly low at 44 h compared to the WT. Strains were grown on a 70:30 plate and the number of total spores present at 44 h was counted under a phase-contrast microscope to calculate sporulation efficiency. The means and standard errors of the means from three biological replicates are shown, with significance being determined by the two-tailed Student *t* test. *, *P* ≤ 0.05. Download FIG S4, TIF file, 0.2 MB. Copyright © 2020 Ahmed et al. 2020 Ahmed et al. This content is distributed under the terms of the Creative Commons Attribution 4.0 International license.

10.1128/mBio.03190-20.5 FIG S5 Verification of the *agr1* complement strains. (A) Genomic DNA from WT, *agr1* mutant, and *agr1* complement strains was used to amplify a region of the *agr1* locus to confirm the presence of the *agr1* locus in these complement strains. (B) Expression of the *agr1* locus in the complement strains was examined using RT-qPCR. The means and standard errors of the means from two replicates are shown. Download FIG S5, TIF file, 1.5 MB. Copyright © 2020 Ahmed et al. 2020 Ahmed et al. This content is distributed under the terms of the Creative Commons Attribution 4.0 International license.

When supernatants from each of the mutants were tested for cytopathic effects, we found very similar levels (<30%) of cell rounding in the C. difficile WT, C. difficile Δ*agrD1*, and C. difficile Δ*agrB1D1* but almost 80% cell rounding when cells were treated with C. difficile Δ*agrB1* supernatant-treated cells (Fig. 5G). Thus, the changes in toxin transcription and protein levels correlate with increased toxic effects in C. difficile Δ*agrB1*.

To demonstrate if the increased toxin production in the C. difficile Δ*agrB1* was due to the loss of *agrB1* gene, in our next set of experiments we examined transcript levels of *tcdA*, *tcdB*, and *tcdR*, as well as protein levels of TcdA and TcdB, in the C. difficile Δ*agrB1*::*BD* complement strain. In C. difficile Δ*agrB1*::*BD*, transcript levels of *tcdA*, *tcdB*, and *tcdR* were significantly decreased (∼3.9-, ∼3.3-, and ∼2.5-fold decreases, respectively) compared to those in C. difficile Δ*agrB1* (Fig. 6A). The C. difficile WT::*BD* strain did not exhibit any significant change in the transcript levels of *tcdA*, *tcdB*, and *tcdR* (Fig. 6A). Additionally, immunoblot analysis of lysates taken from C. difficile Δ*agrB1*::*BD* showed 2.1-fold and 2.24-fold decreases in TcdA and TcdB, respectively, compared to lysates from C. difficile Δ*agrB1* (Fig. 6B). Corresponding TGX stain-free gels that were used to develop this immunoblots are shown in Fig. S3E and F.

### Intracellular AgrD1 accumulates in C. difficile Δ*agrB1*.

**FIG 7:**
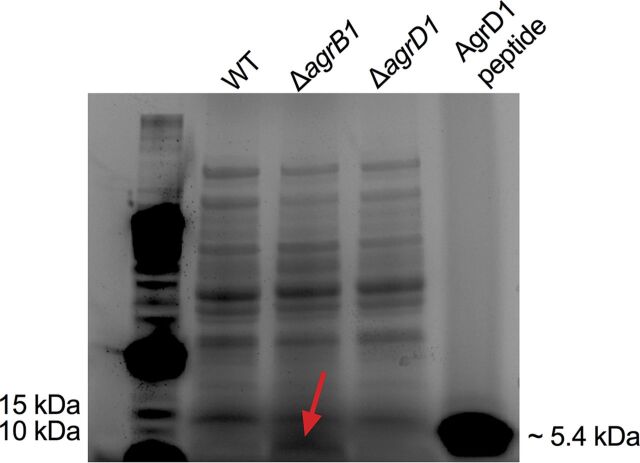
SDS-PAGE analysis of lysates from C. difficile 630 and *agr1* mutants. Lysates were resolved on an SDS-PAGE gradient gel and stained with Coomassie blue. A synthetic AgrD1 peptide was included for comparison. The red arrow indicates the area of increased Coomassie blue staining in C. difficile Δ*agrB1* lysates, which corresponds to the molecular weight of AgrD1. MS/MS analysis of the corresponding band revealed a peptide with a sequence identical to that of AgrD1.

Because several of the observed phenotypes were distinct to C. difficile Δ*agrB1* (Fig. 2B and C and Fig. 5), we were curious to know if the intact AgrD1 peptide accumulated within the cytoplasm of this mutant. To assess this possibility, stationary-phase lysates of C. difficile 630, C. difficile Δ*agrB1*, and C. difficile Δ*agrD1* were prepared and resolved on a 4 to 15% SDS-PAGE gel. The gel was stained with Coomassie blue and then examined for the band corresponding to the size range of AgrD1 (∼5.4 kDa). As shown in the Fig. 7, only C. difficile Δ*agrB1* lysates showed stained protein resolving near the dye front of the gel at approximately the size expected for AgrD1. To confirm the presence of AgrD1, the band was extracted and subjected to in-gel trypsin digestion for mass spectrometry (MS) analysis and tandem MS (MS/MS) sequencing. A synthesized AgrD1 peptide was included as a control and for comparison with the extracted band from the C. difficile Δ*agrB1*. In both cases, analysis of the gel-extracted sample and control peptide revealed a peak of approximately 938.0 *m/z*, which corresponds to that expected for a trypsin-digested AgrD1 peptide (Fig. S6). MS sequencing of the peak identified the sequence as FASSLALSTAILSANSTCPWIIHQPK, which is an exact match to the sequence within AgrD1 (Fig. S6). These data indicate that the AgrD1 peptide may accumulate within the cytoplasm of C. difficile in the absence of AgrB1.

10.1128/mBio.03190-20.6 FIG S6 Mass spectrometry analysis of the band corresponding to the size range of AgrD1 in C. difficile Δ*agrB1*. (A) MS1 spectrum of the AgrD1 corresponding peptide fragments generated after tryptic digestion of the excised bands from the AgrD1 peptide (control) and C. difficile Δ*agrB1*. (B) MS/MS analysis of the suspected peptide from C. difficile Δ*agrB1* found to match to an internal fragment (FASSLALSTAILSANSTCPWIIHQPK) of the AgrD1 peptide. Download FIG S6, TIF file, 1.4 MB. Copyright © 2020 Ahmed et al. 2020 Ahmed et al. This content is distributed under the terms of the Creative Commons Attribution 4.0 International license.

## DISCUSSION

Unlike most AIP systems, the C. difficile Agr1 operon does not encode a definitive two-component signaling system and encodes only the AgrB1 protease and the AgrD1 peptide. The curious absence of AgrC and AgrA led us to explore the Agr1 system and address two questions. First, does C. difficile Agr1 function exclusively as an AIP-quorum sensing system? Second, which regulatory networks are impacted by Agr1? We addressed the first question by developing a Cas9 nickase genome editing system that allowed us to generate deletions in *agrB1* and *agrD1*, as well as a complete deletion of both genes. We predicted that each of the mutants should have the same phenotype if Agr1 is only involved in peptide quorum sensing because in each case the mutant would be unable to produce the AIP. To address the second question, we examined each of the mutants for changes in the expression of key transcriptional regulators related to sporulation, motility, and toxin production. The collective data this study indicate that the C. difficile Agr1 system appears to function in a typical manner as relates to sporulation, but the role of Agr1 in toxin regulation appears to involve mechanisms that do not rely on AgrB.

Using results from the targeted transcript analysis, we suspected the Agr1 system could be involved in sporulation. The absence of AgrB1, AgrD1, or both proteins resulted in a dramatic reduction in sporulation efficiency. And, critically, when supernatants from stationary-phase cultures of C. difficile 630 were applied to the mutants, sporulation was recovered. This indicates that Agr1 does indeed function, under certain circumstances, as a bona fide AIP system, and we suspect that there are a sensor kinase and response regulator likely encoded elsewhere in the genome of C. difficile 630 that relay AIP signals within the cell during sporulation. Earlier studies on Clostridium botulinum used a combination of small interfering RNA (siRNA) and ClosTron technology to either delete or silence expression of AgrB and AgrD individually (6). Similar to what we observed in this study, both AgrB and AgrD were found to be required for effective sporulation in C. botulinum. In addition, deletion of *agrB* in Clostridium perfringens type A was reported by Li and colleagues (11) to effectively eliminate sporulation. These data suggest that C. difficile sporulation is strongly influenced by the Agr1 system in C. difficile 630 and this may be common in other *Clostridia*.

One of the most striking changes we detected was the increase in *tcdR* in C. difficile Δ*agrB1*. Levels of *tcdR* in C. difficile Δ*agrB1* were increased almost 13-fold compared to those in the parental strain and the other two mutants. Importantly, the relative increased levels of *tcdR* transcripts in C. difficile Δ*agrB1* correlated with the increase in TcdA. In contrast, we did not detect changes in transcript levels for *codY* or *ccpA* for any of the mutants. The data from these experiments suggest that TcdR levels increase in the C. difficile Δ*agrB1*, and this could be an important factor in heightened expression of C. difficile toxins in this mutant.

We were particularly interested in the transcriptional and functional changes related to motility because motility-related genes and their products have previously found to be closely tied to toxin production in C. difficile (21). Among the panel of genes analyzed, we found *fliC* transcripts elevated almost 26-fold in C. difficile Δ*agrB1* and 5-fold in C. difficile Δ*agrD1*. Previous work by Aubry and colleagues indicated that a *fliC* mutant in C. difficile 630 Δ*erm* expressed higher levels of TcdA than did the wild type (26). The inverse correlate of this—larger amounts of *fliC* result in smaller amounts of *tcdA*—was not observed during our analysis of the Agr1 mutants. Work by Martin and colleagues also showed that both *fliC* and *tcdA* transcript levels were reduced in an *agrA2* mutant constructed in the epidemic R20297 strain of C. difficile (9). Though the Agr2 system differs from Agr1, these results suggest that a reduction in *fliC* may not always correlate with an increase in *tcdA* expression.

Previous work by Darkoh et al. reported that C. difficile 630 Δ*agrB1D1* expresses smaller amounts of toxins and is attenuated in virulence (10). Likewise, the Agr system has been found to be necessary for toxin expression and virulence in C. perfringens (11, 13, 14). The reasons why the *agrB1D1* mutant generated in our lab did not exhibit the phenotype observed by Darkoh et al. remain unclear. This mutant also exhibited a dramatic reduction in soft-agar motility compared to that of the parent strain and either one of the single mutants. EM analysis also revealed that C. difficile Δ*agrB1D1* lacked detectable flagella. This is similar to what has been reported for the AgrA2 mutant in C. difficile R20291 (9). We detected this phenotype in multiple C. difficile Δ*agrB1D1* mutants generated *de novo*. For reasons unclear to us at this point, each individual C. difficile Δ*agrB1D1* mutant was found to have a single insertion of a guanine (G) in the *fliF* gene at exactly the same location (position 1474). The insertion results in a premature stop codon, with a predicted 25-amino-acid truncation in FliF. This is an unusual occurrence and not typically expected for suppressor mutants, in which one would expect different mutations within the same gene or region. We suspect that this mutation exists at a low level within our parent strain and is enriched when *agrB1D1* is deleted. However, previous studies found that deletion of *fliF* results in decreased toxin expression in C. difficile (26). Therefore, if the truncated and potentially defective form of FliF was completely interfering with the toxin phenotype in the *agrB1D1* mutant, one would still expect to see a decrease in toxin expression.

Eliminating production of the AgrB1 protein resulted in phenotypic changes unlike those observed in the double mutant or in the *agrD1* deletion mutant. mRNA transcripts of *tcdR*, *tcdA*, *fliC*, *flgB*, and *motA* were all increased (*P* < 0.05) in the *agrB1* deletion mutant. We also detected AgrD1 in the cytoplasm of the AgrB1 mutant, suggesting that perhaps the intracellular peptide itself has some previously undescribed intracellular activities. If true, this would represent an entirely new activity that had not been attributed to any Agr system in the past. While this suggests that AgrB1 is dispensable for some activities, it is still likely that Agr1 functions as a traditional AIP quorum sensing system, while also utilizing unprocessed AgrD1 for other activities. Studies with S. aureus found that AgrB processing of AgrD is reversible, reaches equilibrium, and results in accumulation of a nonthiolactone form of AgrD along with the thiolactone form of the peptide (5). Thus, it is possible that at certain points in growth, AgrD1 peptide levels reach a point where the equilibrium shifts to increase intracellular levels of the peptide. Therefore, it is likely that by eliminating AgrB1, we have been able to drive the system in a way that reveals these intracellular effects of AgrD1. Finally, we are unaware of any previous studies showing that AgrB is required for secretion of AgrD in the pathogenic *Clostridia*. In our system, AgrD1 accumulated in the cytoplasm of the *agrB1* mutant, indicating either that the AgrB1-mediated processing of AgrD1 is required for secretion or that AgrB1 itself is directly involved in the secretion of the autoinducing peptide.

In considering which regulatory factors might be impacted by intracellular AgrD1, we were drawn to both SigD and RstA. *sigD* is a known positive regulator of TcdR (27) and thus of toxin expression; therefore, increased *sigD* activity in the *agrB1* mutant could be correlated with observed increased expression of *tcdR* and the toxin genes in this mutant. Transcripts for SigD-dependent genes (*fliC*, *flgB*, and *motA*) are also upregulated in both of these mutants. With regard to RstA, we noticed that the phenotype and transcriptional profiles observed in C. difficile Δ*agrB1* were remarkably similar to those reported for an *rstA* deletion mutant in C. difficile 630 (28). *tcdR*, *tcdA*, and *tcdB* transcripts are increased in both *agrB1* and *rstA* mutants. Neither of the *agrB1* and *rstA* mutants showed a significant change in the expression of *spo0A*, but each exhibited a decrease expression of *sigE* and *sigG*. And although the sporulation phenotype was not unique to C. difficile Δ*agrB1*, the *rstA* mutant is also defective in sporulation. RstA represses its own expression and the expression of *tcdR*, *tcdA*, *tcdB*, and *sigD* by directly binding to the promoter regions of these genes. As shown in Fig. 2C, we detected a small increase in *rstA* transcript levels in C. difficile Δ*agrB1*, but this did not reach the same level of statistical significance as we found for other genes. RstA is a member of the RRNPP protein family, which responds to quorum sensing peptides that are secreted and then reenter the cell to modulate RRNPP protein activity. In the case of RstA, the peptide would be expected to inhibit RstA activity, which would be mimicked by the *rstA* deletion. The regulatory peptide for RstA is currently not known, but given such a strong correlation between the C. difficile Δ*rstA* mutant and C. difficile Δ*agrB1*, the idea that intracellular AgrD1 might repress RstA is intriguing.

## MATERIALS AND METHODS

### Bacterial strains and growth conditions.

C. difficile 630 (GenBank accession no. AM180355) and strains derived from C. difficile 630 were cultured in brain heart infusion medium supplemented with 5 g/liter of yeast extract (BHIS) unless mentioned otherwise. An anaerobic chamber (Coy Laboratory Products) with an atmosphere of 85% N_2_, 10% H_2_, and 5% CO_2_ was used to grow C. difficile anaerobically (29). Thiamphenicol (15 μg/ml) and d-cycloserine (250 μg/ml) were used for counterselection of C. difficile transconjugants against Escherichia coli CA434 after conjugation (30). Complement strains were grown in BHIS medium supplemented with thiamphenicol unless stated otherwise. E. coli was cultured aerobically at 37°C in Luria-Bertani (LB) medium. For selection of plasmids, 12.5 μg/ml of chloramphenicol was used for E. coli NEB10β, and 12.5 μg/ml of chloramphenicol and 50 μg/ml of kanamycin were used for E. coli CA434. B. subtilis (BS49) carrying Tn*916* was grown in BHIS supplemented with chloramphenicol (5 μg/ml) and tetracycline (5 μg/ml). Complemented C. difficile strains were counterselected against BS49 using 50 μg/ml of kanamycin. Strains and plasmids used in this study are listed in Table 1.

**TABLE 1:** List of strains and plasmids used in this study

Strain or plasmid	Relevant description	Source or reference
C. difficile strains		
630	Clinical isolate	ATCC
TMS001	*agrB1* deletion 630 mutant	This study
TMS002	*agrD1* deletion 630 mutant	This study
TMS003	*agrB1D1* deletion 630 mutant	This study
TMS004	C. difficile 630 with an extra copy of *agrB1D1* chromosomally integrated via pTMS006	This study
TMS005	TMS001 with *agrB1D1* chromosomally integrated via pTMS006	This study
TMS006	TMS002 with *agrB1D1* chromosomally integrated via pTMS006	This study
TMS007	TMS003 with *agrB1D1* chromosomally integrated via pTMS006	This study
E. coli strains		
NEB10β	Derivative of DH10B; T1 phage resistant and endonuclease I (*endA1*) deficient	NEB
CA434	Conjugal donor strain HB101 carrying R702	Chain Biotech (32)
NEBTurbo	*recA*^+^ cloning strain to generate plasmid multimers for BS49 uptake	NEB
Bacillus subtilis strain		
BS49	Donor strain for Tn*916* integration into C. difficile	Gift from Joe Sorg (37)
Plasmids		
pMTL84151	E. coli-C. difficile shuttle vector (pCD6, ColE1, *catP*, *tra*)	Chain Biotech (32)
pTMS001	codon-optimized *cas9-*nickase in pMTL84151	This study
pTMS002	*agrB1*-targeted gRNA and homology region in pTMS001	This study
pTMS003	*agrD1*-targeted gRNA and homology region in pTMS001	This study
pTMS004	*agrB1D1*-targeted gRNA and homology region in pTMS001	This study
pMC370	Tn*916* integrational vector (*ermB*, Gram-negative *catP*, *phoZ*)	Gift from Shonna McBride (35)
pTMS005	Removed E. coli *catP* from pMC370 and replaced with clostridial *catP* from pMTL84151	This study
pTMS006	Removed *phoZ* and replaced with *agrB1D1* including 365 bp upstream in pTMS005	This study

### Strain and plasmid constructions.

**(i) Generation of mutants using Cas9 nickase.** The CRISPR-Cas9 nickase vectors used for generating mutant strains were constructed in several steps. The nickase variant of the *Cas9* gene contains a D10A mutation in the RuvC nuclease domain, and only the HNH nuclease domain of Cas9 is functional (31). Therefore, Cas9 nickase introduces a single-strand DNA break at a site targeted by the guide RNA (gRNA) allowing for specific mutations to be created through homologous recombination. The *Cas9* gene from Streptococcus pyogenes was modified (D10A) and codon optimized for C. difficile 630 and synthesized behind the *fdx* promoter containing a ribosomal binding site (RBS) and inserted into the multiple-cloning site (MCS) of pMTL84151 (32) by GenScript Biotech to generate pTMS001. For each mutant, three additional DNA elements were constructed and inserted into pTMS001: left homology donor template, right homology donor template, and a single gRNA. The left and right homology donor templates were designed to flank the coding region of *agrB1* and/or *agrD1*, and these templates were PCR amplified from C. difficile 630 genomic DNA (gDNA) using primers listed in Table S1 (BAL1F to BAL4R) and Q5 high-fidelity PCR polymerase (New England BioLabs [NEB]). The synthetic promoter P4 (33) was chosen to drive the expression of customized gRNAs which produce a single RNA molecule by the fusion of the crRNA (CRISPR RNA that defines genomic target for Cas9 nickase) and tracrRNA (trans-activating CRISPR RNA that acts as a scaffold linking the crRNA to Cas9 nickase) as described previously (34). All P4::gRNA cassettes were synthesized by Integrated DNA Technologies as gBlocks. pTMS001 was linearized using primers BAL5F and BAL5R and Q5 high-fidelity polymerase. Linearized vector was treated with DpnI (New England BioLabs) to remove any remaining vector template following the manufacturer’s protocol. The four pieces (linearized pTMS001, left homology donor template, right homology donor template, and P4::gRNA) were assembled using Gibson assembly following the manufacturer’s protocol (New England BioLabs) to generate the final deletion vector (either pTMS002, pTMS003, or pTMS005) (Fig. S2) and transferred into NEB 10-Beta competent E. coli cells via transformation and plated on LB medium supplemented with chloramphenicol. All plasmids were sequence verified (Oklahoma Medical Research Foundation [OMRF]). Confirmed plasmids were transferred into E. coli CA434 via electroporation and finally to C. difficile 630 by conjugation. Obtained colonies were selectively transferred three more times before screening for desired deletion.

**(ii) Generation of complemented strains.** The vector pTMS005 was constructed by adapting a Tn*916*-containing transcriptional reporter (*phoZ*) system vector, pMC370 (a gift from Shonna McBride) (35). First, pMC370 was linearized using primers BAL11F and BAL11R to remove the Gram-negative *catP* gene, followed by DpnI treatment. The *catP* gene from pMTL84151 (Gram-positive *catP*) was PCR amplified using primers BAL13F and BAL13R. Next, linearized pMC370 and the Gram-positive *catP* gene were assembled via Gibson assembly following the manufacturer’s protocol to generate pTMS005, transferred into NEB 10-Beta competent E. coli cells via transformation, plated onto LB medium plus chloramphenicol, and verified via DNA sequencing. To generate pTMS006, the complete *agr1* locus and the apparent promoter sequences (upstream 365 bp) were amplified using primers BAL14F and BAL14R and then cloned into pTMS005, which was linearized using primers BAL12F and BAL12R, thus excluding *phoZ*. The resulting plasmid was then transferred into NEB 10-Beta competent E. coli cells via transformation, plated onto LB medium with chloramphenicol, and sequence verified. Confirmed vectors were transferred to NEB Turbo competent E. coli cells to generate plasmid multimers and then transferred into Bacillus subtilis BS49 (a gift from Joseph Sorg) (36) via transformation. In accordance with published protocols, BS49 containing Tn*916* was next conjugated with C. difficile to generate corresponding complemented strains (35, 37, 38). Transconjugants were selected for integration of the transposon into the C. difficile chromosome using 15 μg/ml of thiamphenicol and counterselected against BS49 using 50 μg/ml of kanamycin. The *agr1* locus was amplified from complemented strain gDNA using primers BAL15F and BAL15R to verify the presence of the *agr1* locus in these strains (Fig. S5).

### Whole-genome sequencing.

Genomic DNA was extracted from C. difficile WT, Δ*agrB1*, Δ*agrD1*, and Δ*agrB1D1* strains using the Sigma bacterial genomic DNA extraction kit, following the manufacturer’s protocol. The genomic DNA was submitted to the Oklahoma Medical Research Foundation Genomics Core Facility and paired-end sequenced on an Illumina MiSeq. The data were aligned to the reference genome (GenBank accession number AM180355) using Geneious version 10.2.4 (39).

### RNA extraction, cDNA synthesis, and RT‐qPCR.

Overnight C. difficile cultures (optical density [OD] ∼ 1.0) were diluted 1:50 in fresh BHIS medium and incubated at 37°C. Samples for RNA extraction were collected at an OD of 1.0 and diluted into RNAprotect bacterial reagent (Qiagen). Cells were then harvested by centrifugation and stored at −80°C. RNA was extracted using the Direct-zol RNA extraction kit (Zymo Research) followed by Turbo DNase I treatment (Ambion). cDNA was synthesized from 1 μg of RNA using SuperScript IV VILO master mix. cDNA synthesis reaction mixture containing no reverse transcriptase was used as a negative control in subsequent amplifications to confirm the absence of genomic DNA contamination. Quantitative reverse transcription-PCR (RT-qPCR) was performed in triplicate using iTaq Universal SYBR green Supermix (Bio-Rad) on an Applied Biosystems 7500 fast real-time system. Data were analyzed by the comparative cycle threshold method (ΔΔ*C_T_*, where *C_T_* is the threshold cycle) using the constitutively expressed *rpoC* gene to normalize the amount of transcript of the target gene. Samples from at least three independent experiments were included, and results are presented as the mean and the standard error of the mean from each of those experiments. A two-tailed Student *t* test was performed to analyze statistical significance.

### Sporulation assays and phase-contrast microscopy.

Sporulation assay was performed as described previously (40–42). In brief, C. difficile cultures were grown overnight in BHIS medium with 0.1% taurocholate. Overnight cultures were then back diluted in fresh BHIS medium supplemented with 0.1% taurocholate. Mid-exponential-phase cultures were normalized to an OD of 0.5, and 150 μl of normalized culture was then plated on a prereduced 70:30 sporulation agar plate. 70:30 sporulation medium is a mixture of 70% SMC (90 g Bacto Peptone, 5 g protease peptone, 1 g NH_4_SO_4_, 1.5 g Tris base, and 15 g agar per liter) and 30% BHIS medium as described previously (43–45). Thiamphenicol slows the growth of C. difficile culture; therefore, to ensure that all strains were in the same growth phase, thiamphenicol was not added in the 70:30 sporulation agar plate for all strains tested. Cells on 70:30 agar plates were harvested at the desired time points. For phase-contrast microscopy, harvested cells were resuspended in phosphate-buffered saline (PBS) and removed from anaerobic chamber. Cells were pelleted and resuspended in 50 μl of PBS. Eight microliters of the concentrated culture was then applied to a 0.7% agarose pad and phase-contrast microscopy was performed using an Olympus BX51 instrument. At least three fields per strain were obtained to count vegetative cells and spores. Percent sporulation was calculated as [number of spores/(number of vegetative cells + number of spores)] × 100. Three independent experiments were performed to test each strain.

For heat resistance assays, cells harvested from 70:30 plates were resuspended in 1 ml of PBS. For total CFU count, an aliquot of resuspended culture was serially diluted in PBS and plated onto a BHIS agar plate with taurocholate (0.1%). To determine spore count, an aliquot of resuspended culture was heated at 65°C for 25 min using a heat block in the anaerobic chamber, serially diluted, and plated onto a BHIS agar plate with taurocholate (0.1%). CFU were determined after 40 h of incubation in the anaerobic chamber. Percent sporulation was calculated as (number of heat-resistant spores/number of total cells) × 100.

### Sporulation measurements in *agr1* mutants treated with supernatants from C. difficile 630.

Overnight cultures of C. difficile strains were back diluted in fresh BHIS medium and grown to an OD of 1.0. Cultures were centrifuged at 4,000 × *g* for 10 min at 4°C, and the supernatant was filter sterilized using a 0.2-μm syringe filter. An Amicon Ultra centrifugal filter unit (cutoff 10 kDa) was then used to pass filter-sterilized supernatant in order to obtain a flowthrough with small peptides. A total of 500 μl of the prepared supernatant was spread on prereduced 70:30 plates and allowed to dry, and then 150 μl of the exponential-phase C. difficile cultures (wild type and *agr1 mutants*) were spread on those pretreated 70:30 plates. Plates were incubated for 22 h, and spore counting was performed using the heat resistance assay as described above.

### Motility assays.

Motility assays were performed as described previously (46, 47). C. difficile was grown overnight in BHIS medium and back diluted 1:50 in fresh BHIS medium. Growth from exponential phase (OD ∼0 .7) was normalized to an OD of 0.5, and 5-μl volumes of cultures were stab inoculated in overnight-solidified one-half BHI plates with 0.3% agar for swimming motility and spot inoculated on 2-h-solidified one-half BHI plates with 0.3% agar to test swarming motility. The diameter of each growth was measured every 24 h for a span of 5 days. Images were taken using a Bio-Rad ChemiDoc system on day 5. For each strain, three biological replicates were examined as well as three technical replicates for each of these biological replicates.

### Flagellar negative staining.

Overnight cultures of C. difficile were fixed overnight at 4°C in a solution containing 2% paraformaldehyde (EM grade), 2.5% glutaraldehyde (EM grade), and 0.1 M sodium cacodylate buffer (pH 7.2). A 10-μl sample was applied onto 300-mesh, Formvar-coated, glow-discharged copper grids using the single-drop method and allowed to settle on the grid for 3 min. The sample was removed by wicking with filter paper and rinsed twice for 10 s with Nanopure water. In between each wash, water was removed by wicking with filter paper. Next, 10 μl of 4% uranyl acetate in Nanopure water was deposited on the grid for 45 s, and then the staining solution was removed by wicking with filter paper flowed by washing with Nanopure water for 10 s. The grid was allowed to air dry for 60 s, and finally, grids were viewed on a Hitachi H7600 transmission electron microscope at 80 kV equipped with a 2k × 2k AMT digital camera. Described procedures were performed at the Oklahoma Medical Research Foundation Imaging Core, Oklahoma City, OK.

### Western blot analysis.

Overnight C. difficile cultures were diluted 1:50 into fresh BHIS medium and then grown to an OD at 600 nm (OD_600_) of 1.0. These cultures were centrifuged at 4,000 × *g* for 10 min, and the resulting cell pellet was lysed in 2% SDS by bead beating. The crude cell lysates were clarified, and protein concentration was determined by Lowry assay. Twenty micrograms of protein was resolved on 4 to 15% TGX stain-free precast SDS-PAGE gel (Bio-Rad). Before transfer to a polyvinylidene difluoride (PVDF) membrane, total protein was imaged using a Bio-Rad ChemiDoc MP system to confirm equal loading. The membranes were then blocked with 5% milk in wash buffer (Tris-buffered saline, 0.1% Tween 20) and probed overnight with antibody specific to either TcdA (catalog number NB600-1066; Novus Biologicals) or TcdB (catalog number AF6246; R&D Biosystems), followed by washing and incubation with horseradish peroxidase (HRP)-conjugated secondary antibody for 1 h at room temperature. A chemiluminescent enhancement system (catalog number 1705061; Bio-Rad) was used to develop the blots, and visualization was achieved with a Bio-Rad ChemiDoc MP system. Densitometry was analyzed using Image Lab software (Bio-Rad).

### Cytopathic-effect assay.

HeLa cells were seeded in 96-well plates at a density of 1 × 10^4^/well and incubated overnight at 37°C. At 24 h after plating, cells were treated with C. difficile supernatant (OD = 1.0) using dilutions ranging from 1:10 to 1:10^7^. Treated cells were then incubated for 24 h at 37°C. Cytopathic effect (i.e., cell rounding) was then determined by visualizing cell rounding under an Olympus IX51 bright field microscope. At least 2 fields from each technical replicates per treated group were obtained to count total and rounded cells. Percentage of rounded cells were calculated as (number of rounded cells/number of total cells) Amicon Ultra centrifugal filter unit × 100. Two biological replicates were tested for each strain.

### Detection of intracellular AgrD1 peptide.

Overnight C. difficile 630 cultures were back diluted in 50 ml of fresh BHIS medium and grown to an OD of 1.0. These cultures were centrifuged at 4,000 × *g* for 10 min. The resulting cell pellet was resuspended in NP-40 lysis buffer and lysed by bead bursting. The crude cell lysates were clarified, and 20 μl of protein was analyzed by 4 to 15% SDS-PAGE (Bio-Rad) and Coomassie blue staining. The band corresponding to the protein suspected to be AgrD1 was identified by using a Thermo Tribrid Fusion Lumos Orbitrap mass spectrometer at the University of Oklahoma Health Sciences Center (OUHSC) core facility using established in-gel trypsin protocols (48). The full-length AgrD1 peptide used as a control in this experiment was synthesized using LifeTein peptide synthesis services.

### Data availability.

The whole-genome sequencing data were deposited in the NCBI SRA database under BioProject identifier (ID) PRJNA610762.
