# Supplementary figures and images for: Combined and Distinct Roles of Agr Proteins in Clostridioides difficile 630 Sporulation, Motility, and Toxin Production
Source: mBio. 2020 Dec 22;11(6):e03190-20. doi: 10.1128/mBio.03190-20 (PMC8534292; doi:10.1128/mBio.03190-20)

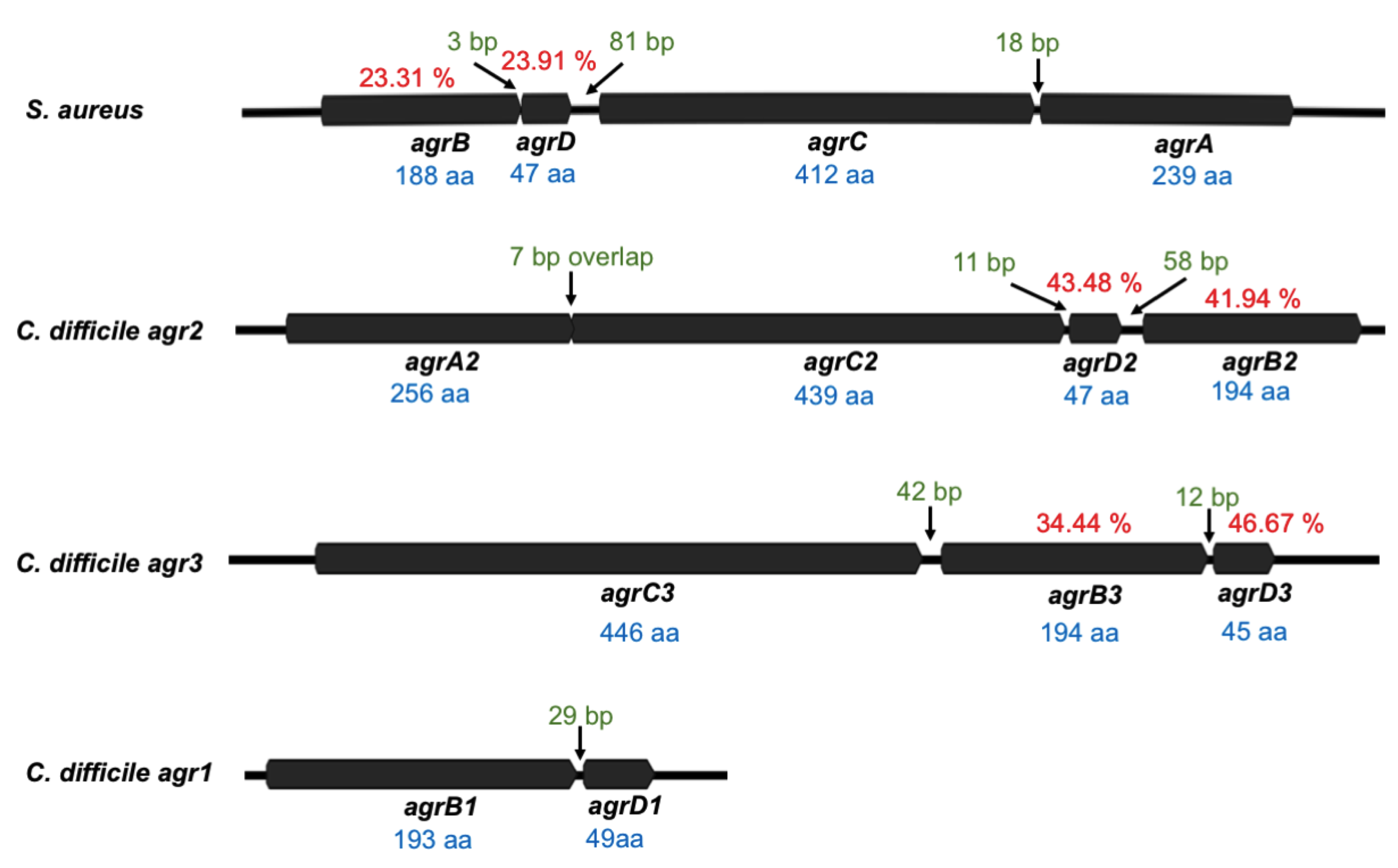

Supplement: FIG S1 [file mbio03190-20-sf001.tif]

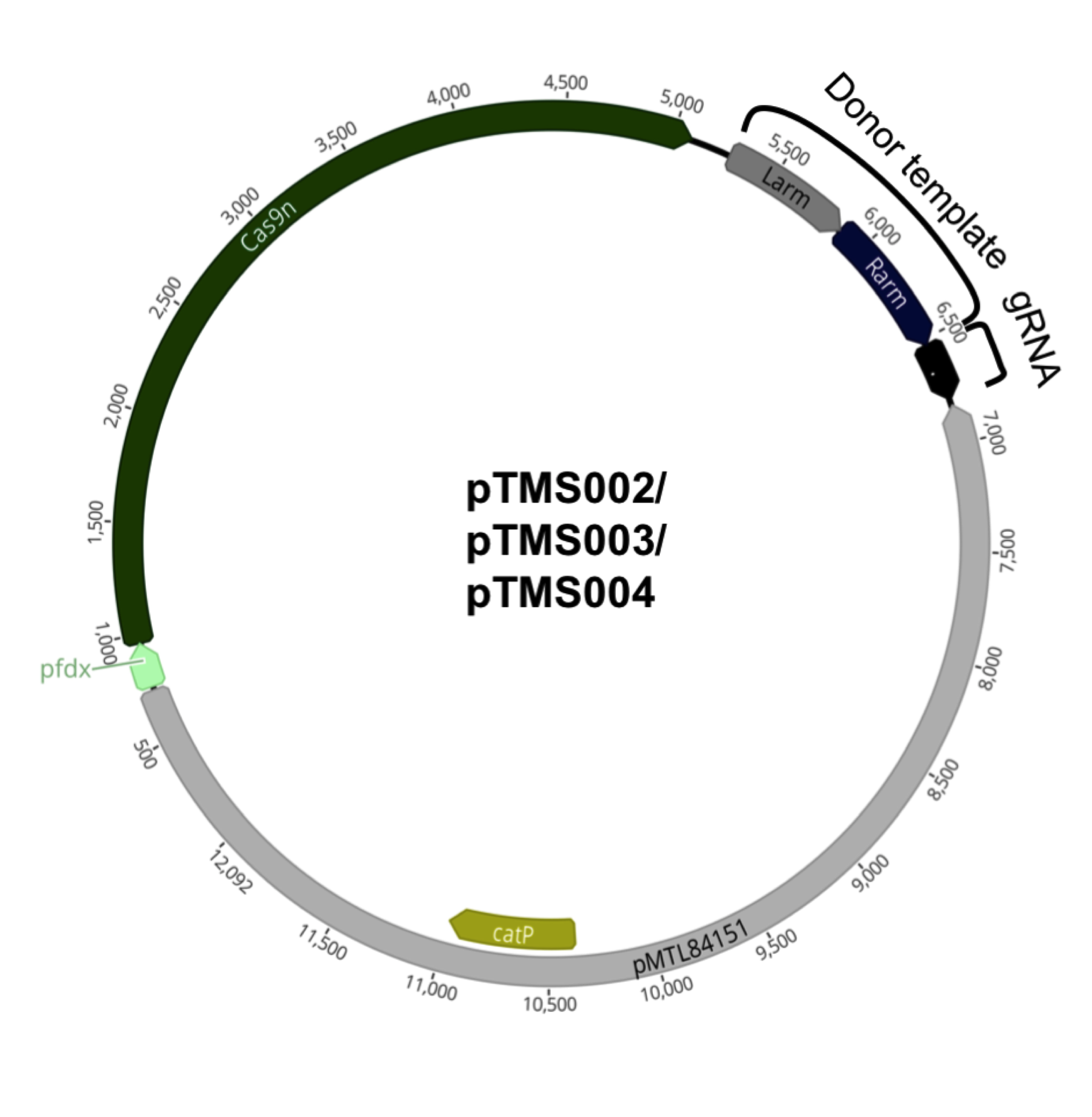

Supplement: FIG S2 [file mbio03190-20-sf002.tif]

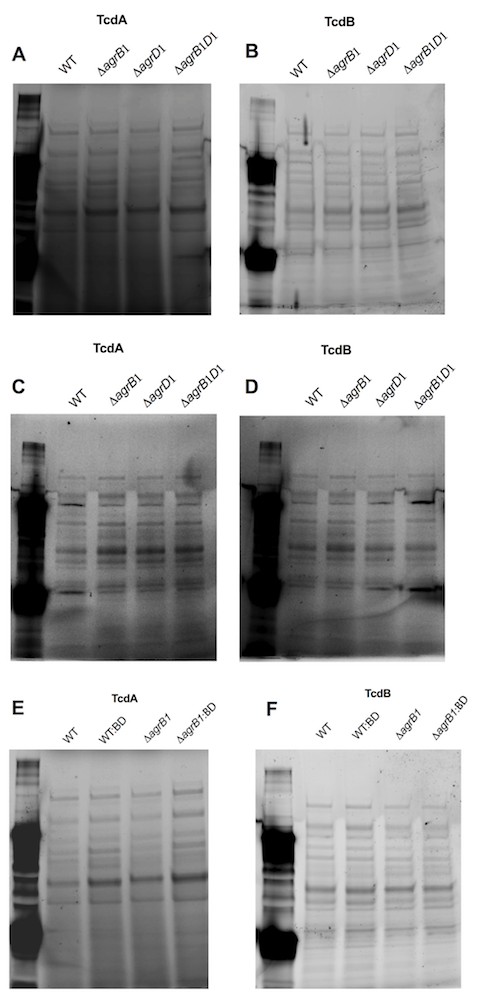

Supplement: FIG S3 [file mbio03190-20-sf003.tif]

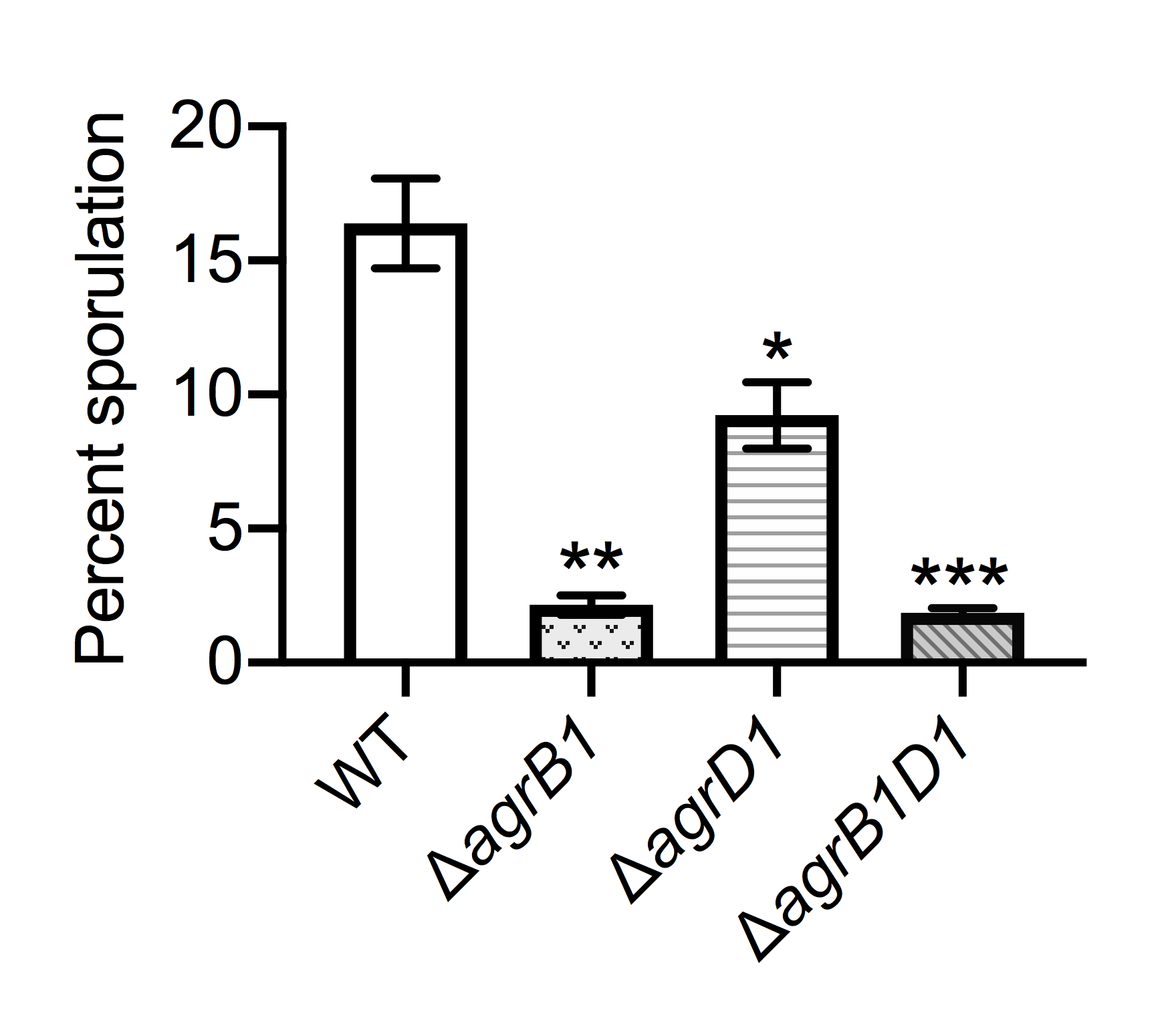

Supplement: FIG S4 [file mbio03190-20-sf004.tif]

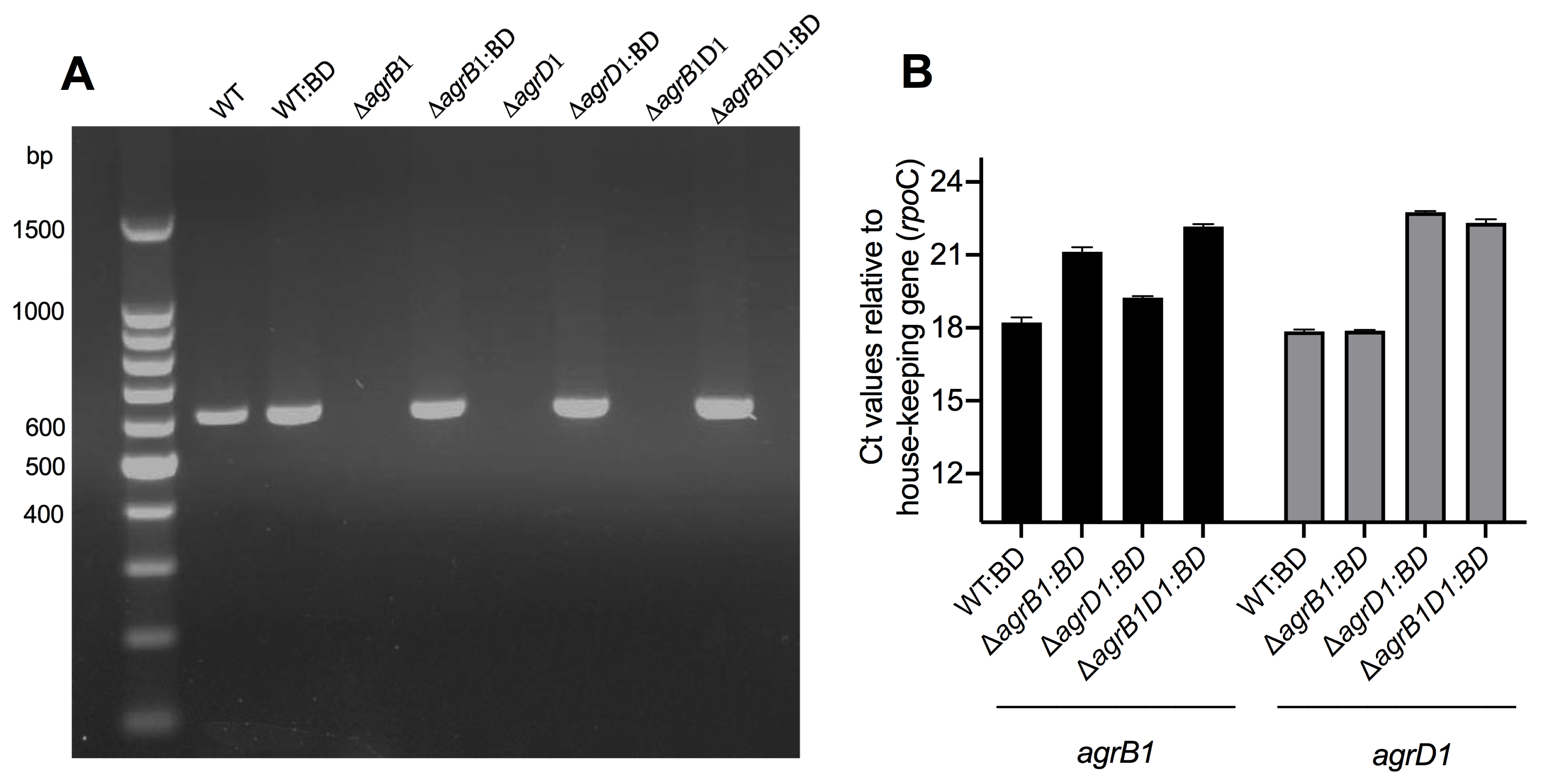

Supplement: FIG S5 [file mbio03190-20-sf005.tif]

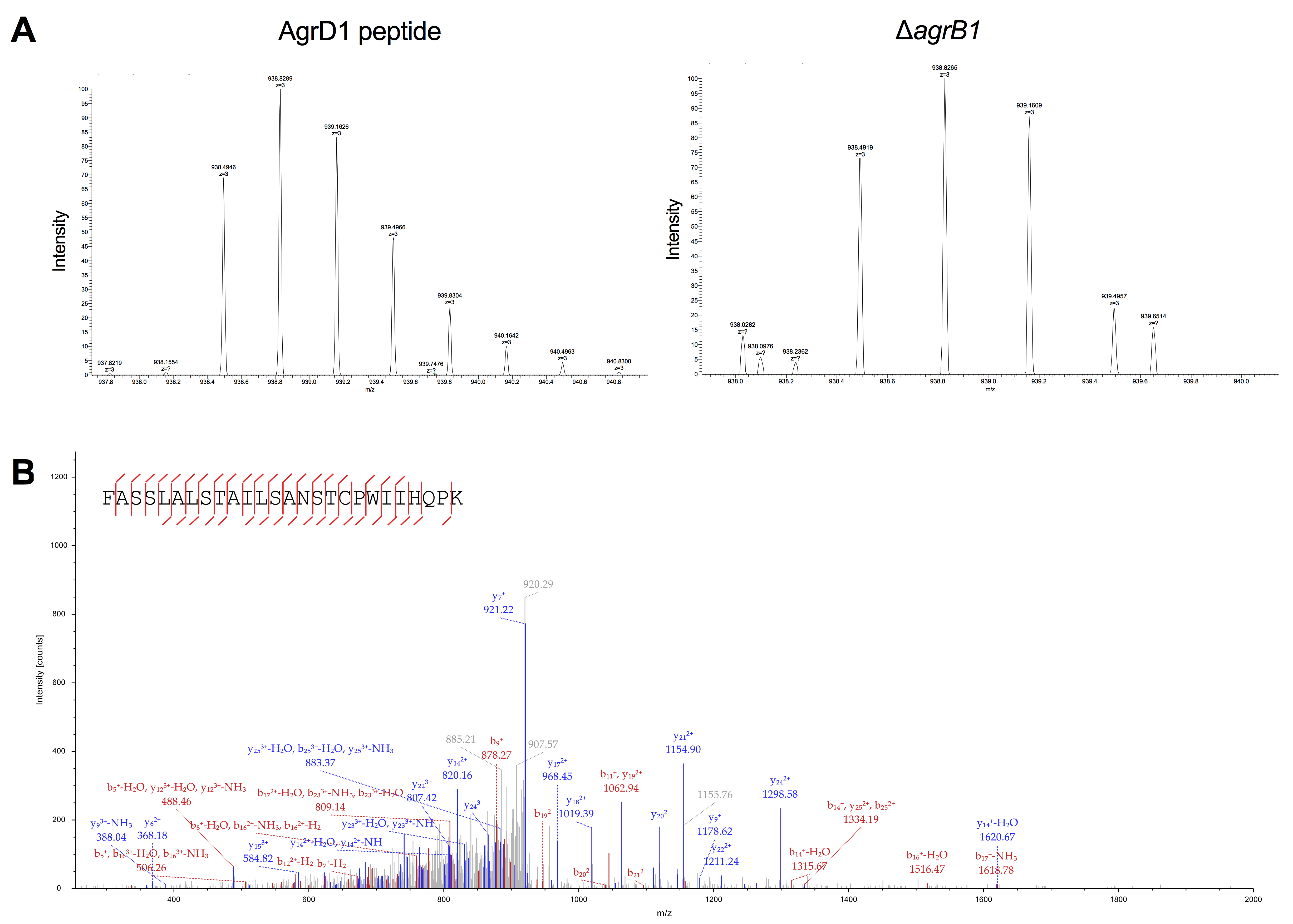

Supplement: FIG S6 [file mbio03190-20-sf006.tif]
