# Supplementary material for: Combined and Distinct Roles of Agr Proteins in Clostridioides difficile 630 Sporulation, Motility, and Toxin Production
Source: mBio. 2020 Dec 22;11(6):e03190-20. doi: 10.1128/mBio.03190-20 (PMC8534292; doi:10.1128/mBio.03190-20)
Supplement: TABLE S1 [file mbio03190-20-st001.pdf]

Table S1. List of primers used in this study.

Prime	Sequences (5' – 3')	Use	Source
BAL1F	ATACATATTTTACGCATATCTTTTAA ACATTTTTACC	To amplify left HDT to flank *agrB1*	This study
BAL1R	TTGCAGCACATCCCCCTTTCGCCAG GCGCTTAAATATGTGAAAG	To amplify left HDT to flank *agrB1*	This study
BAL2F	TTGCTCATCAATTTGTTGCAACGA GACAAATCGTATGCAACATC	To amplify right HDT to flank *agrB1*	This study
BAL2R	TTAAAAGATATGCGTAAAATATGT ATGAGGGAGAGTTAAG	To amplify right HDT to flank *agrB1*	This study
BAL3F	TTGCAGCACATCCCCCTTTCGCCAG AATGACATCTGTTTTAATTTGT	To amplify left HDT to flank *agrD1*	This study
BAL3R	ATA AGT GCT TGT AAA CTT AAC TCT CCC TCA TAC A	To amplify left HDT to flank *agrD1*	This study
BAL4F	GGAGAGTTAAGTTTACAAGCACTT ATTTATACAAAATTATC	To amplify right HDT to flank *agrD1*	This study
BAL4R	TTGCTCATCAATTTGTTGCAACGAG CCTTGAGCTGCAAAACAGTA	To amplify left HDT to flank *agrD1*	This study
BAL5F	CGCCCGCCCTTAAGTCTAAAAATTA GGGGAGATG	To linearize pTMS001	This study
BAL6R	CAGGGTTTTCCCAGTCACGACGTT GTAAAACGAC	To linearize pTMS001	This study

BAL7F	CGTTTTACAACGTCGTGACTGGGAAAACCC	pTMS001/002/003 insert screening primer	This study
BAL7R	CTATTTTTCCCAAATCCTTACATCTCCCCT	pTMS001/002/003 insert screening primer	This study
BAL8F	TGCTTGGAGTCAGTTAGACA	Δ*agrB1* screening primer	This study
BAL8R	ATCAAAGGCTCTCCACCAA	Δ*agrB1* screening primer	This study
BAL9F	AGCGCATTTGCCATGTTTTG	Δ*agrD1* screening primer	This study
BAL9R	TTGCTGCCTTACCAACTTCA	Δ*agrD1* screening primer	This study
BAL10F	AGCCTTTTAACAATGGGGAA	Δ*agrB1D1* screening primer	This study
BAL10R	ACAAAAATGACTTACTCTCCGT	Δ*agrB1D1* screening primer	This study
BAL11F	GATCAACGTCTCATTTTCGCCAAAAGTTGG	pMC370 linearizing primer	This study
BAL11R	TTAAGGCAGTTATTGGTGCCCTTAAACGC	pMC370 linearizing primer	This study

BAL12F	GCATGCACCATTCCTTGC	pTMS005 linearizing primer	This study
BAL12R	GTAGTCGATAGTGGCTCC	pTMS005 linearizing primer	This study
BAL13F	TTAAGGGCACCAATAACTGCCTTAAGGGCAAGTTGAAAAATTCACAAAAATG	To amplify *catP* from pMTL84151	This study
BAL13R	TTTTGGCGAAAATGAGACGTTGATCGTTTAAACTTAGGGTAACAAAAAACACCG	To amplify *catP* from pMTL84151	This study
BAL14F	GCTACTTGGAGCCACTATCGACTACGGCTAAAATATATAGAAAATATATGCAAATAAATAAAATTT	To amplify *agr1* and upstream 365 bp	This study
BAL14R	CGCCGCCGCAAGGAATGGTGCATCTTAATTTGTCTTCTTTAGATTGC	To amplify *agr1* and upstream 365 bp	This study
BAL15F	ACCACCATGCTGTTTATAGGT	Screening primer for complement strains	This study
BAL15R	GATTGCTGATTTCTTTGGGTACTT	Screening primer for complement strains	This study
BAL16F	ACCACCATGCTGTTTATAGGT	*agrB1* qPCR primer	This study
BAL16R	CTGCATGATACCCTCCTGAAA	*agrB1* qPCR primer	This study

BAL17F	TGCTAGCTCATTGGCACTT	*agrD1* qPCR primer	This study
BAL17R	GATTGCTGATTTCTTTGGGTACTT	*agrD1* qPCR primer	This study
BAL18F	GCAGTCACTGGATGGAGAATTA	*tcdA* qPCR primer	This study
BAL18R	AGATGATAGCAGTGTCAGGATTG	*tcdA* qPCR primer	This study
BAL19F	GAAGGATTACCTATAATTGC	*tcdB* qPCR primer	This study
BAL19R	CTGCCATTATACCTATCTTAG	*tcdB* qPCR primer	This study
BAL20F	TTATTAAATCTGTTTCTCCCTCTTCA	*tcdR* qPCR primer	McKee *et al.*, 2013
BAL20R	AGCAAGAAATAACTCAGTAGATGATT	*tcdR* qPCR primer	McKee *et al.*, 2013
BAL21F	GAGCACAAAGGGTATTGCTCTA	*tcdC* qPCR primer	This study
BAL21R	AAATGACCTCCTCATGGTCTTC	*tcdC* qPCR primer	This study
BAL22F	CTCATCTTCTATAACTGAACTGTCTTGAAC	*codY* qPCR primer	Edwards *et al.*, 2016
BAL22R	TTTGATTTACTGGCCGGAGCATTG	*codY* qPCR primer	Edwards *et al.*, 2016
BAL23F	TCTTGTTCAACTATCCATGAAATCATAAC	*ccpA* qPCR primer	Edwards *et al.*, 2016
BAL23R	AAATGGGATAGAAGAGGTTGCTAAA	*ccpA* qPCR primer	Edwards *et al.*, 2016
BAL24F	TTGCAAGAGTTACTTCATTCTGATTT	*rstA* qPCR primer	Edwards *et al.*, 2016

BAL24R	TGTAAGATAGCCTTAGCTTCATCAATA	*rstA* qPCR primer	Edwards *et al*., 2016
BAL25F	GTTGTTATACCAGCTGAAGCCATTA	*fliC* qPCR primer	Edwards *et al*., 2016
BAL26R	TACAAGTTGGAGCAAGTTATGGAAC	*fliC* qPCR primer	Edwards *et al*., 2016
BAL27F	GCATCAATCAATCCAATGACTCCAC	*sigD* qPCR primer	Edwards *et al*., 2016
BAL27R	TGCCTCTTGTAAAGAGTATAGCA	*sigD* qPCR primer	Edwards *et al*., 2016
BAL28F	CTCTAGTTCTAAGATGGACCTTATCTC	*motA* qPCR primer	McKee *et al*., 2013
BAL28R	CAATAGAGAGTGATGTAATGGGAATAGAAG	*motA* qPCR primer	McKee *et al*., 2013
BAL29F	AGGCATAGCATCATTTAGTGTTTC	*flgB* qPCR primer	Edwards *et al*., 2016
BAL29R	CAACTAATCTAAGAAGTCAGACAATAGC	*flgB* qPCR primer	Edwards *et al*., 2016
BAL30F	TTGAGTCTCTTGAACTGGTCTAGG	*spo0A* qPCR primer	Edwards *et al*., 2014
BAL30R	CTCAAAGCGCAATAAATCTAGGAGC	*spo0A* qPCR primer	Edwards *et al*., 2014

BAL31F	TGACTTTACACTTTCATCTGTTTCTAGC	*sigE* qPCR primer	Edwards *et al*., 2014
BAL31R	GGGCAAATATACTTCCTCCTCCAT	*sigE* qPCR primer	Edwards *et al*., 2014
BAL32F	CGCTCCTAACTAGACCTAAATTGC	*sigF* qPCR primer	Edwards *et al*., 2014
BAL32R	GGAAGTAACTGTTGCCAGAGAAGA	*sigF* qPCR primer	Edwards *et al*., 2014
BAL33F	CAAACTGTTGTCTGGCTTCTTC	*sigG* qPCR primer	Edwards *et al*., 2014
BAL33R	GTGGTGTTAATACATCAGAACTTCC	*sigG* qPCR primer	Edwards *et al*., 2014
BAL34F	CCAGTCTCTCCTGGATCAACTA	*rpoC* qPCR primer	McBride & Sonenshein, 2011
BAL34R	CTAGCTGCTCCTATGTCTCACATC	*rpoC* qPCR primer	McBride & Sonenshein, 2011

HDT, homology donor template
