# Supplementary material for: Combined and Distinct Roles of Agr Proteins in Clostridioides difficile 630 Sporulation, Motility, and Toxin Production
Source: mBio. 2020 Dec 22;11(6):e03190-20. doi: 10.1128/mBio.03190-20 (PMC8534292; doi:10.1128/mBio.03190-20)
Supplement: TABLE S2 [file mbio03190-20-st002.pdf]

Table S2. Percent sporulation of 630 WT, *agr1* mutant, and *agr1* complement strains at 22 hour.

**Strain**	**Total viable cells (CFU/ml)**	**Heat resistant spores (CFU/ml)**	**Percent sporulation (%)**
WT	6.1×10^{9}	1.3×10^{9}	20.7
∆*agrB1*	6.3×10^{8}	4.5×10^{7}	0.68
∆*agrD1*	1.5×10^{10}	2.8×10^{7}	0.18
∆*agrB1D1*	4.2×10^{9}	8.5×10^{6}	0.20
WT:BD	1.4×10^{10}	4.6×10^{9}	31.8
∆*agrB1:BD*	1.0×10^{10}	1.4×10^{9}	13.1
∆*agrD1:BD*	9.8×10^{9}	4.4×10^{8}	4.45
∆*agrB1D1:BD*	7.1×10^{9}	6.5×10^{8}	9.2
